# Lipidation of Class IV CdiA Effector Proteins Promotes Target Cell Recognition during Contact-Dependent Growth Inhibition

**DOI:** 10.1128/mBio.02530-21

**Published:** 2021-10-12

**Authors:** Tiffany M. Halvorsen, Fernando Garza-Sánchez, Zachary C. Ruhe, Nicholas L. Bartelli, Nicole A. Chan, Josephine Y. Nguyen, David A. Low, Christopher S. Hayes

**Affiliations:** a Biomolecular Science and Engineering, University of California, Santa Barbara, Santa Barbara, California, USA; b Department of Molecular, Cellular and Developmental Biology, University of California, Santa Barbara, Santa Barbara, California, USA; c Department of Chemistry and Biochemistry, University of California, Santa Barbara, Santa Barbara, California, USA; Massachusetts Institute of Technology

**Keywords:** bacterial competition, toxin-immunity proteins, type V secretion system

## Abstract

Contact-dependent growth inhibition (CDI) systems enable the direct transfer of protein toxins between competing Gram-negative bacteria. CDI^+^ strains produce cell surface CdiA effector proteins that bind specific receptors on neighboring bacteria to initiate toxin delivery. Three classes of CdiA effectors that recognize different outer membrane protein receptors have been characterized in Escherichia coli to date. Here, we describe a fourth effector class that uses the lipopolysaccharide (LPS) core as a receptor to identify target bacteria. Selection for CDI-resistant target cells yielded *waaF* and *waaP* “deep-rough” mutants, which are unable to synthesize the full LPS core. The CDI resistance phenotypes of other *waa* mutants suggest that phosphorylated inner-core heptose residues form a critical CdiA recognition epitope. Class IV *cdi* loci also encode putative lysyl acyltransferases (CdiC) that are homologous to enzymes that lipidate repeats-in-toxin (RTX) cytolysins. We found that catalytically active CdiC is required for full target cell killing activity, and we provide evidence that the acyltransferase appends 3-hydroxydecanoate to a specific Lys residue within the CdiA receptor-binding domain. We propose that the lipid moiety inserts into the hydrophobic leaflet of lipid A to anchor CdiA interactions with the core oligosaccharide. Thus, LPS-binding CDI systems appear to have co-opted an RTX toxin-activating acyltransferase to increase the affinity of CdiA effectors for the target cell outer membrane.

## INTRODUCTION

Bacteria compete for growth niches and other limited resources in densely populated communities. One common competitive strategy entails the direct transfer of toxic effector proteins into neighboring rivals. Antibacterial effectors are deployed though several specialized export pathways, including the type I (1), type IV (2), type V (3), and type VI (4, 5) secretion systems of Gram-negative bacteria. Species of myxobacteria use outer membrane exchange to deliver lipoprotein toxins (6), and Esx-like secretion systems in Gram-positive bacteria have been reported to deliver effectors in a cell contact-dependent manner (7). Direct interbacterial toxin delivery was first described as contact-dependent growth inhibition (CDI) in Escherichia coli EC93, which uses CdiB and CdiA two-partner secretion (TPS) proteins to kill other strains of E. coli (3). Related TPS proteins are found in a variety of Gram-negative proteobacteria, *Fusobacteria*, and *Negativicutes* (8, 9), and CDI activity has been demonstrated in Dickeya dadantii (8), Burkholderia thailandensis (10, 11), Neisseria meningitidis (12), Burkholderia dolosa (13), Burkholderia cepacia (14), Pseudomonas aeruginosa (15, 16), and Acinetobacter baumannii (17, 18). CdiB is an Omp85 family protein that transports the CdiA effector across the outer membrane. CdiA is thought to remain associated with CdiB, and the effector protein forms a filament extending several hundred angstroms from the cell surface (19). CdiA recognizes specific receptors on neighboring bacteria and then delivers its C-terminal toxin domain (CdiA-CT) to inhibit target cell growth. CDI^+^ strains protect themselves from self-intoxication by producing CdiI immunity proteins that bind and inactivate the CdiA-CT. CdiA-CT sequences are extraordinarily variable, and strains of the same species often deploy distinct toxins. CdiI sequences are also highly variable, and together with CdiA-CT domains, they comprise a complex network of polymorphic toxin-immunity protein pairs. Because CdiI proteins neutralize only their cognate CdiA-CT toxins, CDI is thought to mediate interstrain competition for growth niches and other environmental resources. However, these systems also contribute to cooperative group activities. CdiA-receptor binding interactions promote cellular autoaggregation and biofilm formation (20–22). Thus, CDI contributes to fitness by facilitating cooperative interactions between sibling cells, as well as by inhibiting the growth of nonisogenic competitors.

CdiA proteins vary considerably in size and sequence between bacterial species, but all share an architecture with constituent domains arranged from the N to C terminus in the order they function during toxin delivery. A Sec-dependent signal sequence targets CdiA to the periplasm, where CdiB recognizes the N-terminal TPS transport domain and guides the effector across the outer membrane (23, 24). Following the TPS domain is an extensive region of filamentous hemagglutinin 1 (FHA-1) peptide repeats. The FHA-1 repeats fold into a β-helical filament as they emerge from CdiB into the extracellular space. The size of the FHA-1 domain varies between species, with extracellular filaments predicted to extend ∼15 to 100 nm from the cell surface (19). The receptor-binding domain (RBD) forms the distal tip of the filament, where it is positioned to interact with neighboring cells. After export of the RBD, secretion is arrested to retain the C-terminal half of CdiA in the periplasm (19). Export resumes once CdiA engages its receptor, and the FHA-2 domain is deposited onto the target cell, where it becomes embedded within the outer membrane (19). FHA-2 is thought to form a translocation conduit to transfer the toxin-containing CdiA-CT region into the target cell periplasm (19). Once inside the periplasm, the CdiA-CT is cleaved from the effector, and the released fragment hijacks integral membrane proteins to enter the target cell cytoplasm (19, 25, 26).

**FIG 1:**
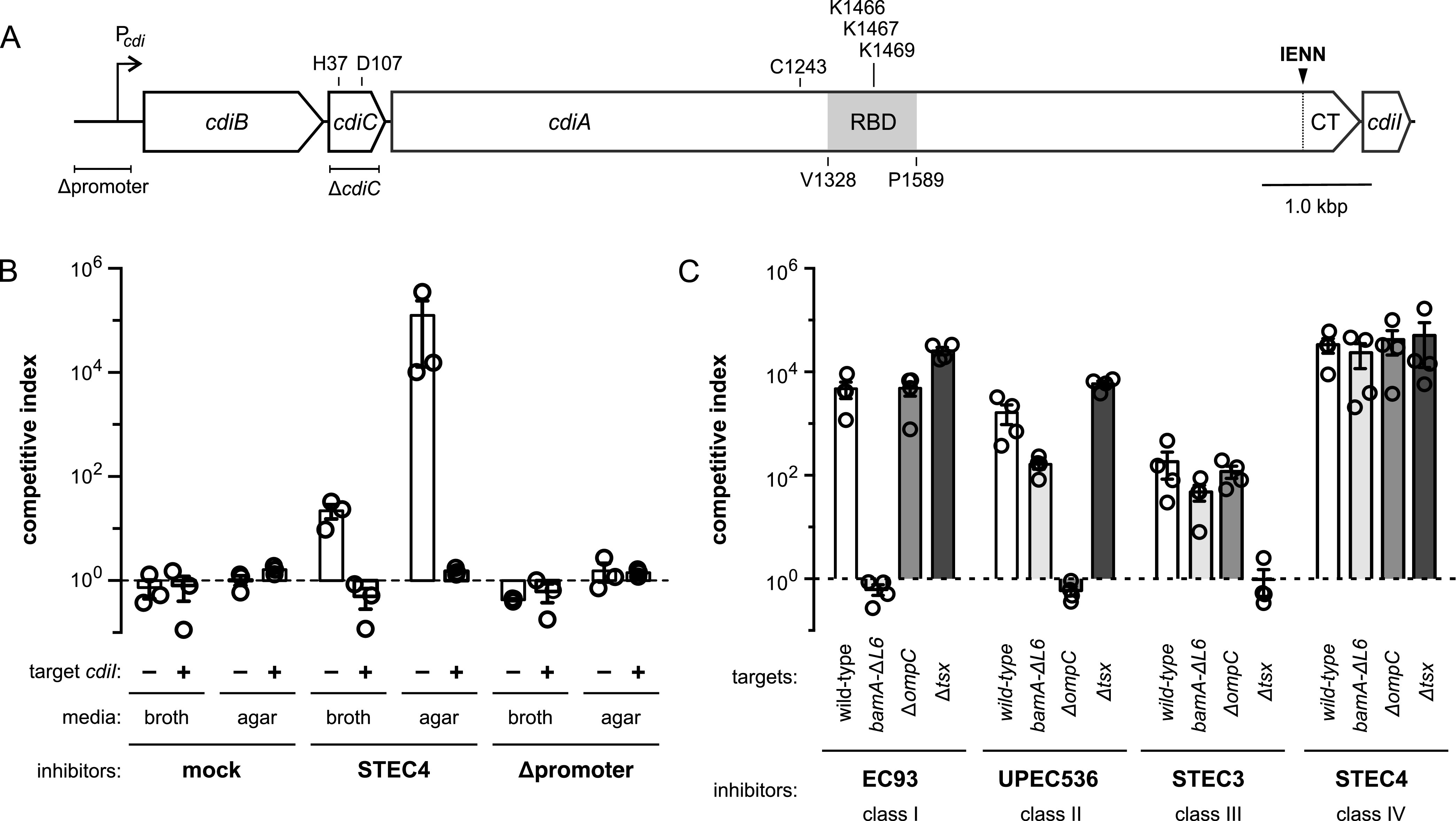
CdiA^STEC4^ recognizes an uncharacterized receptor. (A) Schematic of the class IV *cdiBCAI* locus from E. coli STEC_O31. (B) Inhibitor cells expressing *cdiBCAI*^STEC4^ were seeded at a 1:1 ratio with E. coli *Δwzb* target bacteria for coculture in broth or on solid medium. Where indicated, target cells carried a plasmid-borne copy of *cdiI*^STEC4^. (C) Inhibitor cells expressing class I, II, III, and IV CDI systems were cocultured with E. coli *Δwzb* target bacteria containing the indicated mutations on agar media. The competitive index is the ratio of viable inhibitor to target cells after 3 h. Data are the averages ± SEM from at least three independent experiments.

Three classes of E. coli CdiA have been characterized based on RBD sequences. Class I CdiA^EC93^ from E. coli EC93 recognizes extracellular loops L4 and L6 of BamA (27, 28). CdiA^EC536^ from uropathogenic E. coli 536 is a class II effector that binds to heterotrimeric OmpC/OmpF osmoporins (29, 30). Class III CdiA^STEC3^ from E. coli STEC_O31 uses the outer membrane nucleoside transporter, Tsx, as a receptor (31). Class I, II, and III RBDs only share ∼30% pairwise sequence identity, but the surrounding FHA-1 and FHA-2 peptide repeat domains are highly homologous. This modular architecture allows RBDs to be exchanged between effectors to switch receptor tropism (31). Many E. coli isolates encode a fourth class of CdiA characterized by significantly diverged FHA-1 and RBD regions. Class IV *cdi* loci are also unique in that they contain an additional cistron—that we designate *cdiC*—between the *cdiB* and *cdiA* genes (Fig. 1A). CdiC is homologous to lysyl acyltransferases that activate pore-forming cytolysins of the repeats-in-toxin (RTX) family. Toxin-activating acyltransferases (TAATs) lipidate specific Lys residues within RTX proteins, and the amide-linked acyl chains are required for full cytolytic activity against eukaryotic cells (32). Collectively, these observations suggest that class IV CdiA recognizes an uncharacterized receptor and that its growth-inhibition activity may be modulated through posttranslational lipidation. Here, we show that class IV CdiA effectors use the core oligosaccharide of lipopolysaccharide (LPS) as a receptor and that CdiC modifies a specific Lys residue within the class IV RBD to promote target cell recognition. The sequence surrounding the acylated Lys residue is enriched in aromatic and basic residues, suggesting that this region binds the anionic core of LPS at the aqueous/hydrophobic phase interface. We propose that the 3-hydroxydecanoyl moiety augments this interaction by inserting into the outer leaflet of the target cell outer membrane.

## RESULTS

### CdiA^STEC4^ recognizes an uncharacterized receptor.

E. coli STEC_O31 contains two *cdi* gene clusters that encode class III (CdiA^STEC3^) and class IV (CdiA^STEC4^) effectors (31). CdiA^STEC3^ deploys an EndoU RNase toxin domain that degrades tRNA^Glu^ molecules (19, 33), and the uncharacterized CdiA^STEC4^ protein carries a novel toxin 25 (Ntox25; PF15530) domain recently shown to dissipate the membrane proton gradient (34). To examine CdiA^STEC4^ activity, we cloned the entire *cdiBCAI*^STEC4^ locus onto a plasmid vector (Fig. 1A). E. coli MC1061 cells harboring this plasmid outcompete target bacteria ∼20-fold after 3 h in shaking broth and up to 10^5^-fold when cocultured on agar (Fig. 1B). CdiA^STEC4^ effects this growth advantage, because target bacteria regain competitive fitness when provided with the *cdiI*^STEC4^ immunity gene (Fig. 1B). Moreover, deletion of the predicted *cdi* promoter region from the plasmid construct abrogates inhibition activity (Fig. 1A and B), indicating that the gene cluster is expressed from native regulatory elements. We next tested whether CdiA^STEC4^ utilizes any of the previously identified receptors for CdiA. Target strains carrying *bamA*(*ΔL6*), Δ*ompC*, and Δ*tsx* mutations are specifically resistant to class I CdiA^EC93^, class II CdiA^EC536^, and class III CdiA^STEC3^, respectively (Fig. 1C). However, each mutant strain is inhibited by CdiA^STEC4^ to the same extent as wild-type target cells (Fig. 1C), indicating that class IV CdiA^STEC4^ recognizes an unknown receptor.

### Deep-rough mutants are resistant to CdiA^STEC4^.

**FIG 2:**
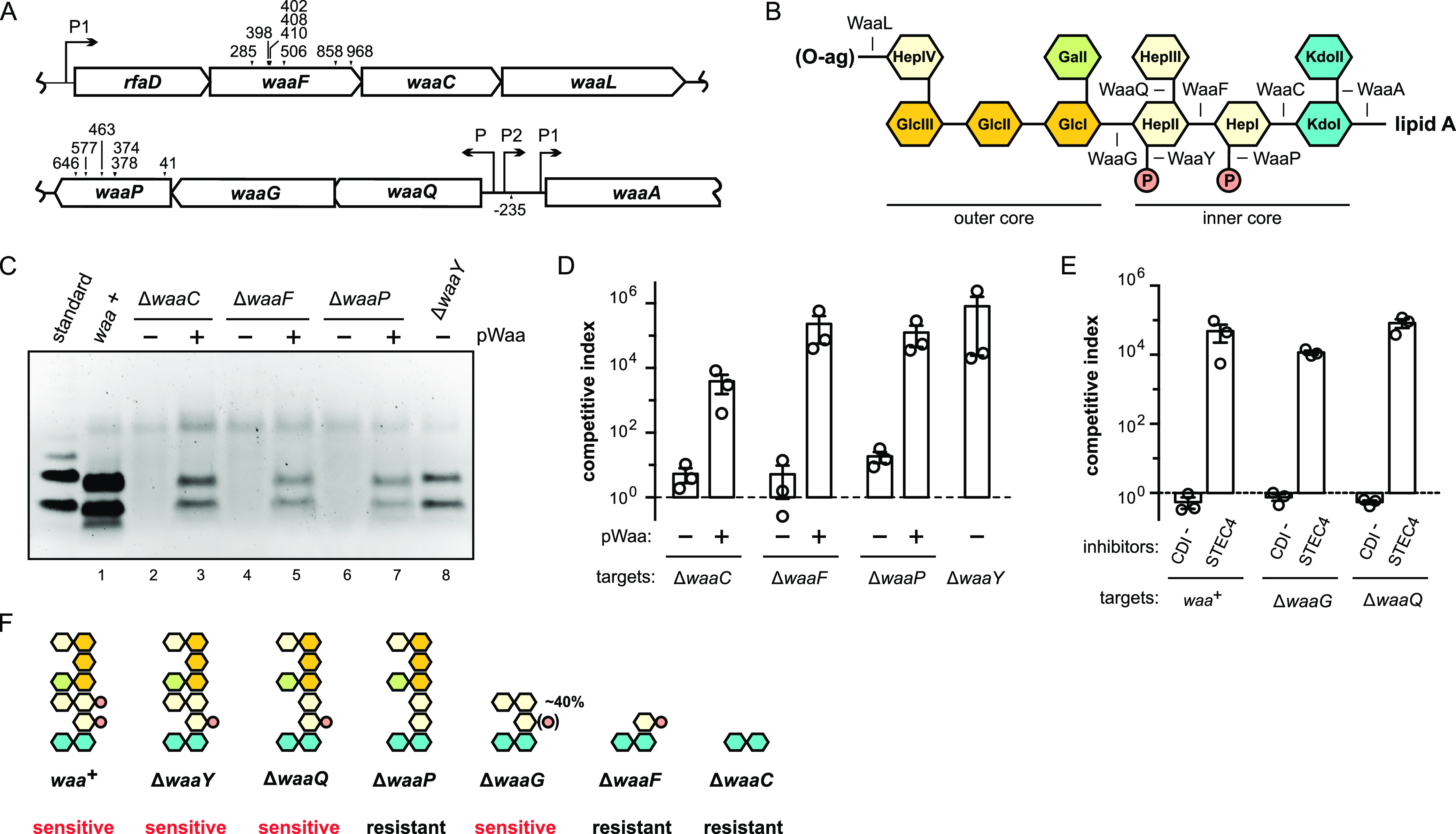
E. coli *waa* mutants are resistant to CdiA^STEC4^. (A) *mariner* transposon insertion sites in the E. coli K-12 *waa* locus. (B) E. coli K-12 LPS core oligosaccharide structure. Assembly steps for each biosynthetic enzyme are indicated. (C) LPS was isolated from E. coli *waa* mutants, resolved by SDS-PAGE, and stained with Pro-Q Emerald LPS stain. Where indicated, mutant strains were complemented with plasmid-borne copies of wild-type *waa* genes. The LPS standard (from E. coli serotype O55:B5) was provided in the stain kit. (D) CdiA^STEC4^-expressing inhibitor cells were cultured on agar media with E. coli Δ*wzb* target bacteria carrying the indicated *waa* alleles. Competitive indices are the averages ± SEM from three independent experiments. (E) CdiA^STEC4^-expressing and mock (CDI^–^) inhibitor were cultured on agar media with E. coli Δ*wzb* target bacteria carrying the indicated *waa* alleles. Competitive indices are the averages ± SEM from three independent experiments. (F) Core oligosaccharide structures and CDI^R^ phenotypes for the *Δwaa* mutants examined in this study.

To identify the receptor for CdiA^STEC4^, we selected CDI-resistant (CDI^R^) mutants following *mariner* transposon mutagenesis, reasoning that disruption of the receptor gene should protect target cells from growth inhibition. We initially identified insertions in *acrB*, which encodes a multidrug efflux pump that is localized to the cytoplasmic membrane (35, 36). Because AcrB is not exposed on the cell surface, it cannot serve as the receptor for CdiA^STEC4^, although it could be hijacked for toxin transport across the cytoplasmic membrane as has been found for other CDI systems (27, 34). To avoid the isolation of additional *acrB* mutants, we repeated the selection with target bacteria carrying multiple plasmid-borne copies of *acrB*. All of the CDI^R^ mutants obtained from the latter selection contain transposon insertions in the *waa* locus, which encodes enzymes that synthesize the core oligosaccharide of lipopolysaccharide (LPS) (37). These included eight independent insertions in *waaF*, six in *waaP*, and a single insertion in the intergenic region between *waaA* and *waaQ* (Fig. 2A). WaaA is an essential enzyme that transfers two 3-deoxy-*d-manno*-octulonsanic acid (KDO) residues to the lipid IV precursor of lipid A (Fig. 2B) (38). WaaF transfers an l-*glycero*-d*-manno*-heptose (Hep) residue II to the inner core (39), and WaaP is a kinase that phosphorylates heptose I (HepI) (Fig. 2B) (40, 41). WaaP activity is also required for subsequent phosphorylation of HepII and addition of HepIII to the inner core (41). These CDI^R^ isolates are predicted to be classical deep-rough mutants, which have altered cell surface properties that lead to phage resistance and increased susceptibility to hydrophobic compounds (42). These results suggest that the LPS core may be the receptor for CdiA^STEC4^. If this model is correct, then *waaC* mutants should also be CDI^R^, because WaaC is the HepI transferase that acts prior to WaaF and WaaP during core biosynthesis (Fig. 2B). To confirm the role of the *waa* loci in CDI resistance, we constructed Δ*waaF*, *ΔwaaP*, and Δ*waaC* deletion strains for further analyses. LPS extracted from these mutants is difficult to detect using Pro-Q fluorescent dye, but core synthesis is restored to each strain through complementation with the respective *waa* genes (Fig. 2C). Moreover, each deletion mutant is resistant to CdiA^STEC4^, and CDI sensitivity is restored by complementation (Fig. 2D). Thus, the *waaF*, *waaP*, and *waaC* genes are required for intoxication by CdiA^STEC4^.

Although the LPS core is necessary for CdiA^STEC4^ intoxication, CDI resistance could be the result of envelope stress responses that are induced by *waa* mutations. For example, deep-rough mutants upregulate the production of capsular polysaccharide (40), which is known to block CdiA-receptor interactions (27). However, capsule cannot account for resistance here, because all *waa* alleles were evaluated in a capsule-deficient Δ*wzb* background. Deep-rough mutants also induce the σ^E^ envelope-stress regulon, which leads to the synthesis of small regulatory RNAs that decrease outer membrane protein (OMP) production (43, 44). Therefore, CdiA^STEC4^ resistance could reflect the downregulation of an unidentified OMP receptor. To explore this possibility, we examined Δ*waaF* target cells for resistance to CdiA effectors that use known OMPs as receptors. The Δ*waaF* mutation provides some resistance to class I, II, and III effectors, but these target cells are still inhibited 30- to 200-fold during coculture (see Fig. S1A in the supplemental material). This effect could be due to decreased receptor expression, because immunoblotting showed lower levels of BamA and OmpC in Δ*waaF* mutants than in *waa^+^* cells (Fig. S1B). BamA and OmpC are also reduced in Δ*waaC* mutants (Fig. S1B). In contrast, Δ*waaP* cells appear to have wild-type OMP levels, though we detected an increase in BamA degradation products similar to the *ΔwaaF* and Δ*waaC* backgrounds (Fig. S1B). Given that Δ*waaF* cells are only partially resistant to OMP-targeting effectors, and that OMP levels are minimally perturbed in CdiA^STEC4^-resistant Δ*waaP* mutants, we conclude that CdiA^STEC4^ recognizes the LPS core as a receptor.

10.1128/mBio.02530-21.1 FIG S1 E. coli deep-rough mutants are partially resistant to outer membrane protein (OMP)-targeting CdiA effectors. (A) Inhibitor strains expressing the indicated CDI systems were cocultured at a 1:1 ratio with E. coli CH7175 (*waa^+^*) or CH13816 (*ΔwaaF*) target cells on LB agar. The competitive index is the ratio of viable inhibitor to target cells after 3 h. Data are the averages ± SEM from three independent experiments. (B) Total urea-soluble protein was isolated from the indicated *waa* backgrounds for immunoblot analysis using polyclonal antibodies to OmpC and BamA. The arrow indicates an apparent BamA degradation product that accumulates in deep-rough mutant backgroundsFIG S1, PDF file, 0.5 MB. Copyright © 2021 Halvorsen et al. 2021 Halvorsen et al. https://creativecommons.org/licenses/by/4.0/ This content is distributed under the terms of the Creative Commons Attribution 4.0 International license.

The predicted LPS structures of Δ*waaF*, *ΔwaaC*, and Δ*waaP* mutants suggest that CdiA^STEC4^ binds to the inner core region. To test this model, we examined the resistance profiles of Δ*waaG*, *ΔwaaQ*, and Δ*waaY* target cells. WaaG and WaaQ transfer glucose (GlcI) and HepIII residues (respectively) to the core, and WaaY is the kinase that phosphorylates HepII (Fig. 2B) (41). Notably, WaaY activity is dependent upon both WaaG and WaaQ, and HepI phosphorylation is reduced by ∼60% in *waaG* mutants (45). Δ*waaG*, *ΔwaaQ*, and Δ*waaY* mutants are all inhibited by CdiA^STEC4^ to the same extent as *waa*^+^ cells on solid media (Fig. 2D and E), indicating that HepIII and the outer core are not important for recognition. Because Δ*waaP* and Δ*waaY* cells differ only in HepI phosphorylation (Fig. 2F), this modified residue is likely a key binding epitope. HepII also appears to be critical, because the terminal HepI-phosphate residue on *ΔwaaF* cells is not sufficient for recognition by CdiA^STEC4^ (Fig. 2D and F).

### RBD^STEC4^ binds cells in a *waa*-dependent manner.

**FIG 3:**
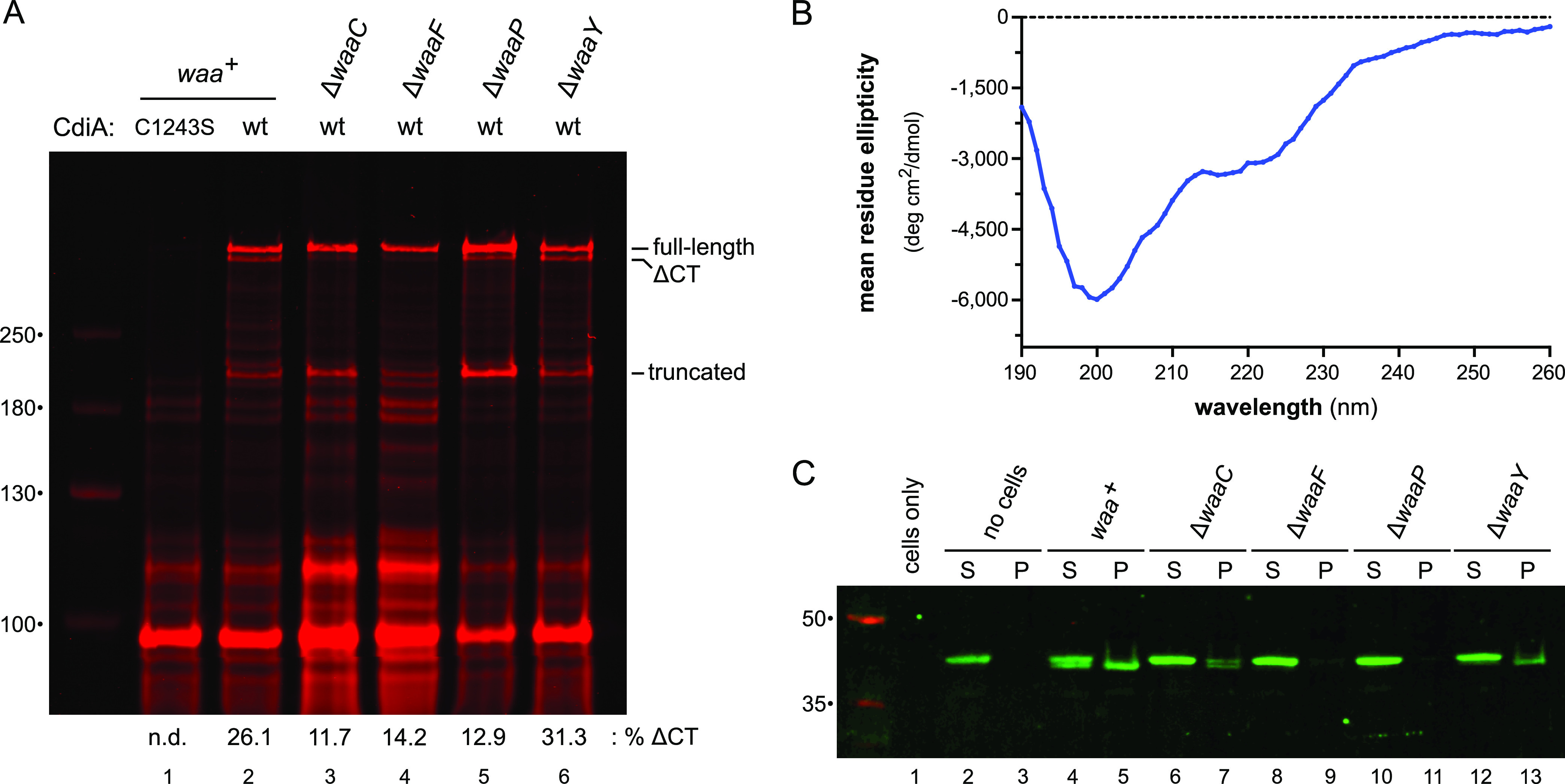
RBD^STEC4^ binds cells in a *waa*-dependent manner. (A) E. coli strains producing wild-type or Cys1243Ser CdiA^STEC4^ were incubated with IRDye680-maleimide, and urea-soluble protein was extracted for SDS-PAGE and fluorimetry. The migration positions for full-length, ΔCT processed, and truncated CdiA^STEC4^ are indicated. Dye fluorescence was quantified for full-length and ΔCT forms of CdiA^STEC4^, and the percentage of CT processed chains is reported below the fluorogram. (B) Circular-dichroism spectrum of purified RBD^STEC4^-His_6_. (C) E. coli *waa* mutant cells were incubated with purified RBD^STEC4^-His_6_ and centrifuged into supernatant (S) and cell pellet (P) fractions. Proteins were extracted and analyzed by SDS-PAGE and anti-His_6_ immunoblotting.

Because CDI^+^ inhibitors readily deliver toxin into sibling cells, ΔCT processed forms of CdiA typically accumulate in inhibitor strain monocultures (19, 22). We reasoned that if LPS is required for target cell recognition, then CT processing should be diminished when CdiA^STEC4^ is produced in CDI-resistant Δ*waa* backgrounds. To detect cell surface CdiA^STEC4^, we used a membrane-impermeative, maleimide-conjugated fluorescent dye to label an endogenous Cys residue (Cys1243) within the extracellular FHA-1 domain (Fig. 1A). SDS-PAGE and fluorimaging showed that CdiA^STEC4^ is produced in both full-length (∼300 kDa) and truncated (∼200 kDa) forms (Fig. 3A, lane 2), similar to previously characterized class I and III effectors (19, 22). These labeled proteins correspond to CdiA^STEC4^ chains, because the Cys1243Ser substitution variant—which has the same growth inhibition activity as the wild-type effector (Fig. S2)—does not react with maleimide-conjugated dye under these conditions (Fig. 3A, lane 1). We also detected a cleaved species that migrated as expected for the ΔCT processed form (Fig. 3A, lane 2). Quantification of the full-length and ΔCT forms suggests that ∼26% of the CdiA^STEC4^ chains undergo CT processing associated with toxin delivery. In contrast, CT processing is reduced to about 12 to 14% when CdiA^STEC4^ is produced in *ΔwaaC* and Δ*waaF* cells (Fig. 3A, lanes 3 and 4). We also noted a general increase in the labeling of other proteins in the latter samples, presumably because *waaC* and *waaF* mutants have leaky outer membranes that allow more dye to enter the cell. CT processing is diminished to ∼13% in the Δ*waaP* background (Fig. 3A, lane 5) but is quantitatively similar between Δ*waaY* and *waa^+^* cells (Fig. 3A, lanes 2 and 6). Taken together with the genetic data, these results suggest that CdiA^STEC4^ uses the core oligosaccharide of LPS as a receptor.

10.1128/mBio.02530-21.2 FIG S2 The Cys1243Ser substitution has no effect CdiA^STEC4^ growth inhibition activity. Inhibitor cells expressing wild-type CdiA^STEC4^ or the Cys1243Ser variant were cocultured at a 1:1 ratio with E. coli CH7175 (*waa^+^ Δwzb*) target cells on LB agar. The competitive index is the ratio of viable inhibitor to target cells after 3 h. Data are the averages ± SEM from four independent experiments. Download FIG S2, PDF file, 0.1 MB. Copyright © 2021 Halvorsen et al. 2021 Halvorsen et al. https://creativecommons.org/licenses/by/4.0/ This content is distributed under the terms of the Creative Commons Attribution 4.0 International license.

Sequence alignments indicate that the central portion of CdiA^STEC4^ from residues ∼1300 to 1600 likely corresponds to the RBD (Fig. 1A and Fig. S3). To test this region for receptor binding function, we appended a C-terminal His_6_ tag to residues Val1269 to Pro1589 (which encompass the predicted RBD^STEC4^ and an N-terminal FHA-1 repeat) and purified the protein for cell binding assays. The resulting RBD^STEC4^ fragment is soluble, though the isolated domain appears to be largely unstructured based on its circular-dichroism spectrum, which exhibits a prominent lobe of negative ellipticity centered at ∼200 nm (Fig. 3B). When incubated with *waa*^+^ cells, the RBD^STEC4^ construct is cleaved near its N terminus, and a significant proportion of this processed form remains associated with cells after centrifugation and washing with phosphate buffer (Fig. 3C, lanes 4 and 5). N-terminal cleavage is reduced with the Δ*waaC* mutant, and there is a concomitant decrease in the cell pellet fraction (Fig. 3C, lanes 6 and 7). This effect is more pronounced with Δ*waaF* and Δ*waaP* mutants, which do not interact with purified RBD^STEC4^ (Fig. 3C, lanes 8, 9, 10, and 11). In contrast, CDI-sensitive E. coli *ΔwaaY* mutants appears to bind RBD^STEC4^, though not to the same extent as *waa^+^* cells (Fig. 3C, lanes 12 and 13). In principle, N-terminal processing could convert RBD^STEC4^ into an aggregation-prone form that precipitates during centrifugation. To explore this possibility, we isolated processed RBD^STEC4^ from *waa^+^* cells using Ni^2+^ affinity chromatography and then recentrifuged the protein at 199,000 × *g* to assess solubility. This analysis showed that neither the unprocessed nor the processed form of RBD^STEC4^ pellets during centrifugation (Fig. S4, lanes 1, 2, 3, and 4). Taken together, these results strongly suggest that the central region of CdiA^STEC4^ binds directly to the LPS core.

10.1128/mBio.02530-21.3 FIG S3 Alignment of class I CdiA^EC93^ and class IV CdiA^STEC4^ effector proteins. The amino acid sequences of CdiA^EC93^ (AAZ57198.1) and CdiA^STEC4^ (WP_001081258.1) were aligned using Clustal Omega at http://www.uniprot.org. Domains and peptide motifs are outlined as determined by the NCBI Conserved Domains sequence analysis site. Pink boldface indicates the Sec-dependent signal peptide, green indicates the hemagglutinin activity domain (Pfam: PF05860), blue indicates FHA-1 peptide repeats (Pfam: PF05594), orange indicates FHA-2 peptide repeats (PF13332), yellow indicates the pre-toxin-VENN domain (PF04829), and purple indicates the variable CdiA-CT toxins. The receptor-binding domain of CdiA^EC93^ is shown in black boldface. CdiA^STEC4^ residues Cys1243 and Lys1467 are rendered in red bold font. Download FIG S3, PDF file, 0.07 MB. Copyright © 2021 Halvorsen et al. 2021 Halvorsen et al. https://creativecommons.org/licenses/by/4.0/ This content is distributed under the terms of the Creative Commons Attribution 4.0 International license.

10.1128/mBio.02530-21.4 FIG S4 RBD^STEC4^-His_6_ remains soluble after cell-dependent N-terminal proteolytic cleavage. Unlipidated (no CdiC) and lipidated (+ CdiC) RBD^STEC4^-His_6_ were incubated with E. coli *waa^+^* cells to induce N-terminal processing, and then the processed proteins were reisolated by Ni^2+^ affinity chromatography under denaturing conditions. After buffer exchange into sodium phosphate, processed and unprocessed proteins were centrifuged at 199,000 × *g* for 5 min into supernatant (S) and precipitate (P) fractions for SDS-PAGE analysis. Download FIG S4, PDF file, 0.6 MB. Copyright © 2021 Halvorsen et al. 2021 Halvorsen et al. https://creativecommons.org/licenses/by/4.0/ This content is distributed under the terms of the Creative Commons Attribution 4.0 International license.

### Polymeric O antigen shields receptors from CdiA.

**FIG 4:**
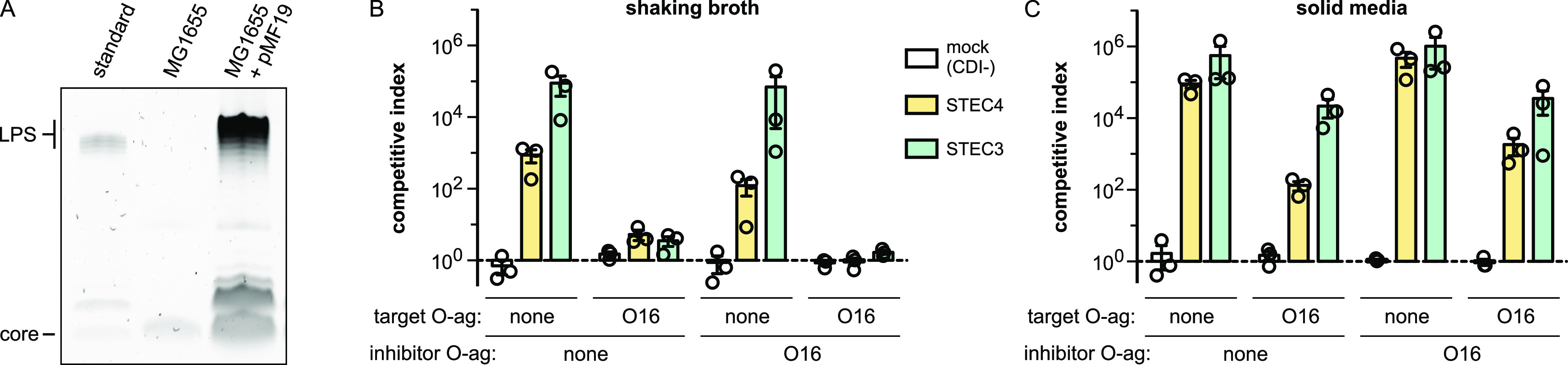
Polymeric O antigen shields CDI receptors. (A) LPS was isolated from the indicated E. coli MG1655 strains, resolved by SDS-PAGE, and stained with Pro-Q Emerald LPS stain. The LPS standard (from E. coli serotype O55:B5) was provided in the stain kit. (B) E. coli MG1655 cells expressing either CdiA^STEC3^ or CdiA^STEC4^ were cocultured at a 1:1 ratio with E. coli MG1655 target bacteria in broth. Where indicated, inhibitor and/or target strains carried plasmid pMF19 to restore O16 antigen (O-ag) synthesis. (C) The inhibitor and target cell strains from panel B were cocultured at a 1:1 ratio on agar media. Competitive indices are the averages ± SEM from three independent experiments.

Most wild isolates of E. coli carry O-antigen polymers linked to the outer core, but domesticated E. coli K-12 strains lack the polysaccharide due to mutations that block its biosynthesis (46). Because CdiA^STEC4^ binds to the LPS core, we asked whether O antigen influences target cell recognition. We first restored O-antigen production in E. coli MG1655 using plasmid-borne *wbbL*—which encodes a rhamnosyl transferase required for O16 antigen synthesis (Fig. 4A) (47, 48)—and then used the complemented strain as a target in competition cocultures. Strikingly, O16^+^ target cells are almost completely resistant to CdiA^STEC4^-mediated growth inhibition in broth cocultures (Fig. 4B). This protective effect is not specific to CdiA^STEC4^, because O16^+^ targets are also resistant to inhibition by class III CdiA^STEC3^ in shaking broth (Fig. 4B). Given that *cdi* genes are found in many wild E. coli isolates, we reasoned that O antigen cannot pose an insurmountable barrier to CdiA. Indeed, O16^+^ target bacteria are inhibited by both CdiA^STEC3^ and CdiA^STEC4^ during competition cocultures on solid media (Fig. 4C). When produced in inhibitor cells, O antigen reduces CdiA^STEC4^ inhibition activity ∼10-fold in shaking broth (Fig. 4B) but appears to increase inhibition activity somewhat on solid media (Fig. 4C). The same trend was observed with inhibitor cells that deploy class III CdiA^STEC3^ (Fig. 4B and C). Thus, O antigen on target cells can shield receptors from CdiA, but the polymer has a more modest effect when present on the surface of inhibitor bacteria.

### CdiC promotes CdiA^STEC4^ growth inhibition activity.

**FIG 5:**
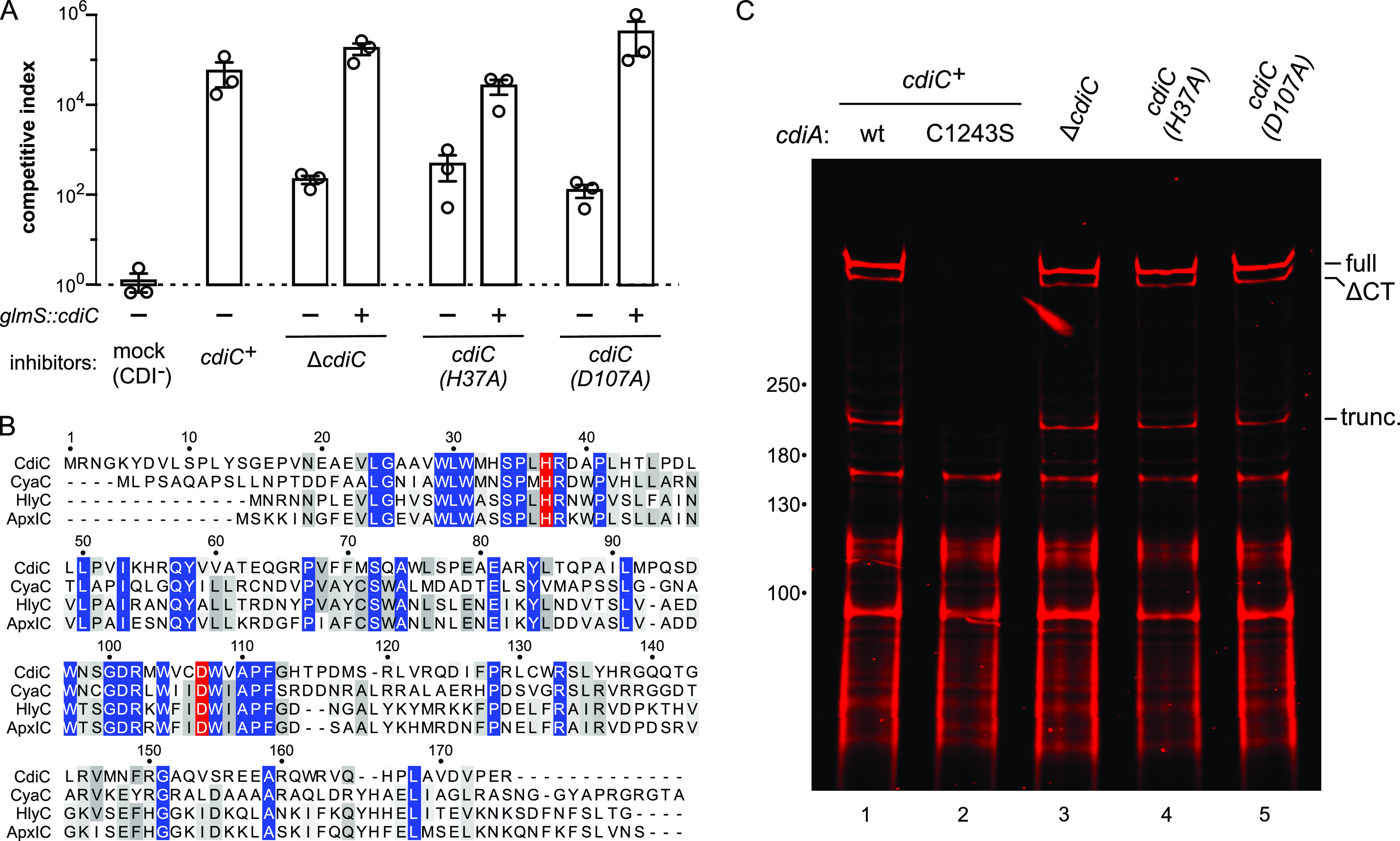
CdiC promotes CdiA^STEC4^ growth inhibition activity. (A) Inhibitor strains carrying the indicated *cdiC* alleles were cocultured at a 1:1 ratio with Δ*wzb* target bacteria on agar media. Where indicated, inhibitor strains were complemented with wild-type *cdiC* integrated at the *glmS* locus. Competitive indices are the averages ± SEM from three independent experiments. (B) Alignment of CdiC^STEC4^ with characterized RTX toxin-activating acyltransferases. Catalytic residues are highlighted in red. (C) E. coli strains expressing CdiA^STEC4^ in the indicated *cdiC* backgrounds were incubated with IRDye680-maleimide, and urea-soluble protein was extracted for SDS-PAGE analysis and fluorimetry. Migration positions for full-length, ΔCT processed, and truncated CdiA^STEC4^ are indicated. wt, wild type.

We next examined the role of CdiC in CDI activity and found that an in-frame *cdiC* deletion reduces growth inhibition ∼100-fold relative to *cdiC^+^* inhibitor cells (Fig. 1A and 5A). This defect is not due to transcriptional polarity on the downstream *cdiA* gene because growth inhibition activity is restored to wild-type levels when *cdiC* is expressed in *trans* from the chromosomal *glmS* locus (Fig. 5A). Alignment with characterized TAAT family members suggests that CdiC residues His37 and Asp107 are important for catalysis (Fig. 5B), and inhibitor strains that express *cdiC*(H37A) and *cdiC*(D107A) missense alleles phenocopy the Δ*cdiC* deletion mutant (Fig. 5A). In principle, CdiC could promote CdiA^STEC4^ export or stabilize the effector protein. However, labeling with extracellular maleimide-dye showed that CdiA^STEC4^ proteins from the Δ*cdiC*, *cdiC*(H37A) and *cdiC*(D107A) constructs are indistinguishable from that produced by the wild-type *cdiC^+^* plasmid (Fig. 5C, compare lanes 1, 3, 4, and 5). Therefore, CdiC activity contributes to target cell killing, but mutations that inactivate the acyltransferase have no obvious effect on CdiA^STEC4^ biogenesis.

### CdiC acylates RBD^STEC4^ with 3-hydroxydecanoate.

**FIG 6:**
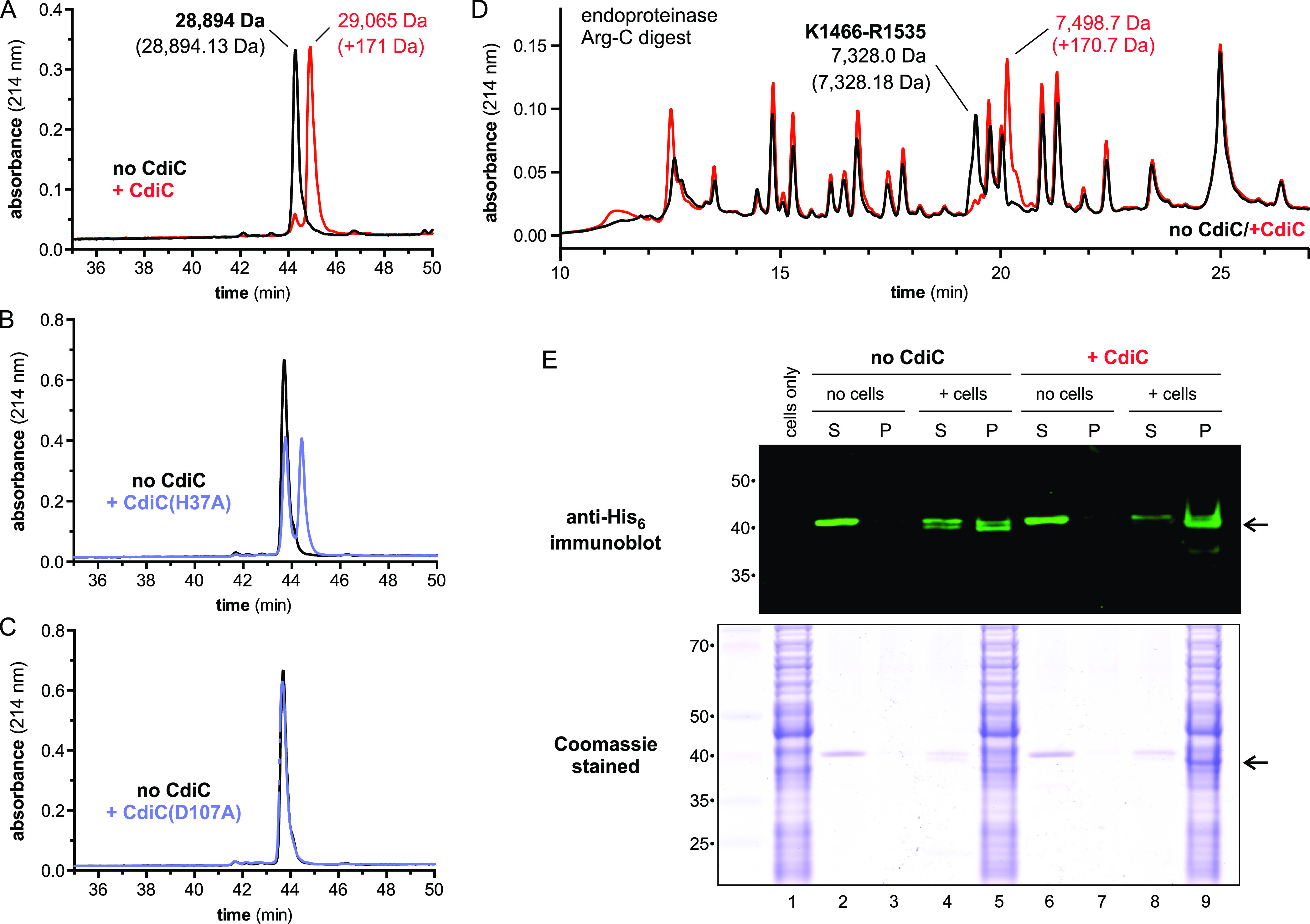
CdiC modifies the RBD of CdiA^STEC4^. (A) His_6_-tagged RBD^STEC4^ was produced with and without CdiC and then purified by Ni^2+^ affinity chromatography for reverse-phase HPLC analyses. Masses were measured by electrospray ionization-mass spectrometry (ESI-MS), and the predicted mass for the unmodified domain is given in parentheses. (B) HPLC analysis of His_6_-tagged RBD^STEC4^ produced with CdiC(H37A). (C) HPLC analysis of His_6_-tagged RBD^STEC4^ produced with CdiC(D107A). (D) Unmodified (black) and modified (red) RBD^STEC4^ was digested with endoproteinase Arg-C and analyzed by reverse-phase HPLC. ESI-MS indicates that the modified peptide corresponds to Lys1466 to Arg1535 of CdiA^STEC4^. (E) E. coli *waa*^+^ cells were incubated with unlipidated or lipidated RBD^STEC4^-His_6_ and centrifuged into supernatant (S) and cell pellet (P) fractions. Proteins were extracted and analyzed by SDS-PAGE and anti-His_6_ immunoblotting. Arrows indicate the cleaved form of RBD^STEC4^ that preferentially associates with cells.

Given that class IV CdiA effectors are encoded adjacent to *cdiC*, we reasoned that the acyltransferase likely modifies the RBD to promote interactions with LPS. To test this hypothesis, we produced CdiC together with a minimal His_6_-tagged RBD^STEC4^ construct (Val1328 to Pro1589) in E. coli cells and purified the domain for biochemical analyses. Reverse-phase high-performance liquid chromatography (RP-HPLC) revealed that RBD^STEC4^ elutes later in acetonitrile gradients when coproduced with CdiC (Fig. 6A), consistent with the addition of a hydrophobic moiety. Furthermore, the mass of RBD^STEC4^ increases by ∼171 Da when coproduced with CdiC. In contrast, RBD^STEC4^ modification is reduced significantly when coproduced with CdiC(H37A) (Fig. 6B), and the domain is not modified by CdiC(D107A) (Fig. 6C). We then used endoproteinase Arg-C peptide mapping to identify the modified peptide(s) by RP-HPLC. Only one peptide fragment, corresponding to residues Lys1466 to Arg1535 of CdiA^STEC4^, was altered in the elution profiles, and its mass increased by ∼171 Da as a result of coproduction with CdiC (Fig. 6D). This shift is most consistent with 3-hydroxydecanoate, which is predicted to increase peptide mass by 170.3 Da. Acylation also appears to increase cell binding affinity, because there is an ∼6-fold increase in lipidated domain recovery from E. coli *waa^+^* cell pellets compared to reactions with unmodified RBD^STEC4^ (Fig. 6E, compare lanes 5 and 9). Because the lipidated, processed form of RBD^STEC4^ does not precipitate at high relative centrifugal forces in the absence of cells (Fig. S4, lanes 7 and 8), these data suggest that the lipid moiety promotes receptor-binding function.

**FIG 7:**
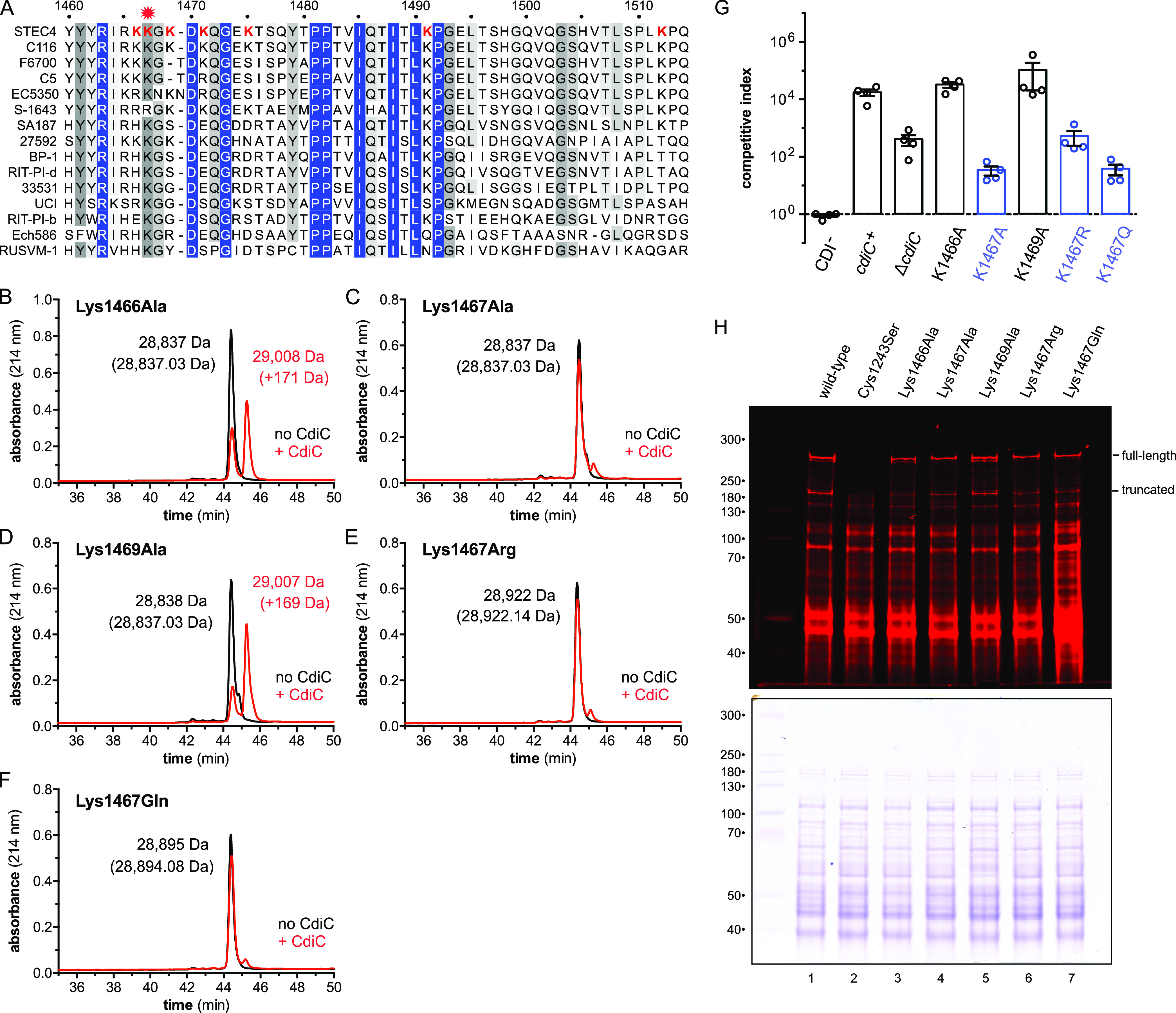
CdiA^STEC4^ residues Lys1467 is acylated by CdiC. (A) RBDs from predicted class IV CdiA proteins of enterobacteria. Lys residues within the modified peptide are indicated in red font. See Fig. S5 for the full alignment and information on bacterial species and accession numbers. (B to F) His_6_-tagged RBD^STEC4^ variants containing Lys1466Ala (B), Lys1467Ala (C), Lys1469Ala (D), Lys1467Arg (E), and Lys1467Gln (F) substitutions were produced with CdiC, and modification was monitored by reverse-phase HPLC. Masses were measured by ESI-MS, and the predicted mass for each unmodified domain is given in parentheses. (G) Inhibitor cells expressing the indicated CdiA^STEC4^ variants were cocultured at a 1:1 ratio with target bacteria on agar media. Competitive indices are the averages ± SEM from three independent experiments. (H) E. coli strains producing wild-type or Cys1243Ser CdiA^STEC4^ were incubated with IRDye680-maleimide, and urea-soluble protein was extracted for SDS-PAGE and fluorimetry. The migration positions for full-length and truncated CdiA^STEC4^ are indicated. After fluorimetry scanning, the gel was stained with Coomassie blue (lower portion).

The modified Arg-C peptide contains seven Lys residues that could potentially be acylated. Alignment with closely related class IV RBDs from enterobacteria reveals that none of these residues is invariant, though Lys1467 is conserved in 14 of the 15 proteins examined (Fig. 7A and Fig. S5). We also noted that Lys1469 is within a Gly-Lys motif recognized by HlyC and CyaC acyltransferases (32). Therefore, we generated Ala substitutions of residues Lys1466, Lys1467, and Lys1469 in the context of the RBD^STEC4^ construct and monitored lipidation using RP-HPLC. The Lys1466Ala substitution significantly reduces modification (Fig. 7B), and the Lys1467Ala mutation completely abrogates modification (Fig. 7C). The Lys1469Ala substitution has only a minor effect on domain modification (Fig. 7D). These substitutions were also incorporated into full-length CdiA^STEC4^ and tested for growth inhibition activity in competition cocultures. The Lys1466Ala and Lys1469Ala variants have the same activity as wild-type CdiA^STEC4^, but inhibitors that deploy the Lys1467Ala variant are less potent than those that lack CdiC altogether (Fig. 7G). The latter result suggests that the Lys1467 side chain may contribute to target cell recognition independent of acylation. Therefore, we also tested Lys1467Arg and Lys1467Gln variants, which cannot be modified by CdiC (Fig. 7E and F). The Lys1467Arg substitution phenocopies the Δ*cdiC* mutation in competition cocultures (Fig. 7G), suggesting that a positively charged residue at this position promotes toxin delivery in the absence of acylation. The Lys1467Gln effector supports the same low inhibition activity as the Lys1467A variant (Fig. 7G). To ensure that these substitutions do not adversely affect export and/or stability, we labeled the CdiA^STEC4^ variants with extracellular dye and confirmed that each is produced at the same level as the wild-type effector (Fig. 7H). Together, these results indicate that acylated Lys1467 contributes significantly to target cell recognition.

10.1128/mBio.02530-21.5 FIG S5 Alignment of enterobacterial class IV RBDs. Predicted class IV effectors were identified by PSI-BLAST searches using CdiA^STEC4^ residues Val1328 to Pro1601 as the query. Hits from enterobacteria were selected for alignment with Clustal Omega and the results rendered using Jalview. Protein sequences are from the following bacterial strains (NCBI reference identifiers in parentheses): E. coli STEC_O31 (WP_001081258.1), E. coli C116 (WP_059337603.1), E. coli F6700 (WP_077784734.1), E. coli C5 (WP_073521113.1), Escherichia sp. strain MOD1-EC5350 (WP_105289418.1), Salmonella enterica serovar Macclesfield strain S-1643 (WP_088731624.1), Enterobacter sp. strain SA187 (WP_083580967.1), Serratia liquefaciens ATCC 27592 (WP_020828638.1), Superficieibacter electus BP-1 (WP_103750823.1), Klebsiella sp. strain RIT-PI-d (WP_049840269.1), Raoultella planticola ATCC 33531 (WP_032700063.1), Enterobacter kobei UCI 24 (WP_032668685.1), *Pantoea* sp. strain RIT-PI-b (WP_049850665.1), Dickeya zeae Ech586 (ACZ78808.1), and Edwardsiella ictaluri RUSVM-1 (WP_081166522.1). The position corresponding to acylated residue Lys1467 in CdiA^STEC4^ is indicated with a red star. Download FIG S5, PDF file, 1.2 MB. Copyright © 2021 Halvorsen et al. 2021 Halvorsen et al. https://creativecommons.org/licenses/by/4.0/ This content is distributed under the terms of the Creative Commons Attribution 4.0 International license.

## DISCUSSION

Our results show that CdiA^STEC4^ from E. coli STEC_O31 uses the LPS core oligosaccharide as a receptor to identify target bacteria. The recognition of LPS during CDI is likely widespread, because other predicted effectors from *Enterobacterales*, *Pseudomonadales*, *Burkholderiales*, and *Negativicutes* carry RBD sequences that are homologous to CdiA^STEC4^ (Fig. S5 and S6). Moreover, another unrelated class of CdiA from *Burkholderia* species also appears to recognize LPS, because B. thailandensis mutants lacking a putative LPS glycosyltransferase encoded by BTH_I0986 are resistant to these effectors (49). The BTH_I0986 gene product is ∼42% identical to LgtG, which transfers α-Glu to the inner core HepI residue of Neisseria gonorrhoeae (50). Although the core structure has not been determined for B. thailandensis, other *Burkholderia* species all contain α-Glu residues linked to HepI (51), raising the possibility that BTH_I0986 produces a CdiA-binding epitope through inner core glucosylation. In contrast to *Burkholderia*, E. coli LPS biosynthesis is well characterized and core structures are known for the *waa* mutants examined in this study. Correlation of mutant core structures with their CDI^R^ phenotypes suggests that HepII and phosphorylated HepI are critical binding determinants for CdiA^STEC4^. Colicin N recognizes a similar overlapping epitope in the LPS core. Sharma et al. found that Δ*waaC*, *ΔwaaF*, *ΔwaaP*, and Δ*waaG* mutants are all resistant to colicin N intoxication (52), and biophysical studies show that this toxin’s RBD interacts directly with GlcI, HepIII, and multiple phosphoryl groups in the inner core (53). LPS is also commonly exploited as a receptor by bacteriophages, and several coliphages use the core oligosaccharide to infect E. coli cells (54, 55). Moreover, O antigen is known to block some phages from gaining access to their inner core receptors (56), akin to the CDI^R^ phenotype it confers in broth coculture. This receptor-shielding phenomenon can also protect E. coli cells from colicin intoxication (57, 58). However, O antigen has only a modest influence on CDI when cells are grown on solid media, suggesting that CdiA filaments readily penetrate the polysaccharide layer of target cells in structured communities. These observations indicate that CDI mainly provides a competitive advantage in densely populated biofilms, consistent with reports that *cdi* expression promotes biofilm formation in several bacterial species (20–22, 59–61).

10.1128/mBio.02530-21.6 FIG S6 Alignment of predicted class IV RBDs. Predicted class IV effectors were identified by PSI-BLAST searches using CdiA^STEC4^ residues Val1328 to Pro1601 as the query. Hits from diverse phyla were selected for alignment with Clustal Omega and the results rendered using Jalview. Protein sequences are from the following bacterial strains (NCBI reference identifiers in parentheses): E. coli STEC_O31 (WP_001081258.1), Klebsiella sp. strain RIT-PI-d (WP_049840269.1), Pseudomonas sp. strain RIT357 (WP_032887927.1), Pseudomonas fluorescens E24 (WP_078827814.1), Ralstonia solanacearum GMI100 (WP_011004362.1), Pseudomonas gingeri F1001 (WP_177143639.1), Methylomusa anaerophila MMFC1 (WP_126305891.1), Herbaspirillum huttiense NFYY 53159 (WP_134221496.1), *Roseateles* sp. strain YR242 (WP_092949725.1), *Sporomusaceae* bacterium FL31 (GBG57802.1), *Orbus* sp. strain IPMB12 (WP_166917331.1), Trinickia symbiotica JPY-366 (WP_107149766.1), Rhodoferax sediminis CHu59-6-5 (WP_142817537.1), and Vogesella mureinivorans 389 (WP_147695851.1). Lys residues corresponding to Lys1467 of CdiA^STEC4^ are marked by a red star and rendered in red font. Strains in blue font do not contain *cdiC* in their *cdi* gene clusters. Download FIG S6, PDF file, 1.2 MB. Copyright © 2021 Halvorsen et al. 2021 Halvorsen et al. https://creativecommons.org/licenses/by/4.0/ This content is distributed under the terms of the Creative Commons Attribution 4.0 International license.

E. coli class IV *cdi* gene clusters encode lysyl acyltransferases related to enzymes that lipidate RTX cytolysins. RTX proteins are pore-forming toxins and include important virulence factors like adenylate cyclase (CyaA) from Bordetella pertussis and α-hemolysin (HlyA) of uropathogenic E. coli strains. CyaA and HlyA are initially synthesized as inactive protoxins that must be lipidated by CyaC and HlyC acyltransferases (respectively) for full cytolytic activity (32). Protoxin activation also depends on acyl-acyl carrier protein (ACP), which serves as the high-energy lipid donor (62). Biochemical and structural studies indicate that TAAT catalysis is mediated by a conserved His/Asp dyad that corresponds to His37 and Asp107 in CdiC (63–65). Worsham and coworkers first proposed that TAAT reactions proceed through an acyl-enzyme intermediate, whereby the active-site His residue accepts the lipid before transfer to the protoxin (66). A more recent model postulates that the acyltransferase, acyl-ACP, and protoxin form a ternary complex for direct lipid transfer (63). In the direct-attack mechanism, the Asp residue abstracts a proton from the protoxin Lys residue to promote its nucleophilic attack on the acyl-ACP thioester, and the His residue protonates the ACP thiolate-leaving group. We found that substitutions in the CdiC catalytic dyad mimic the Δ*cdiC* null phenotype in competition cocultures, but CdiC(H37A) retains significant activity when overproduced with its substrate. Residual activity has also been reported for the analogous His24Ala variant of ApxC (63). These observations are inconsistent with the original “covalent catalysis” model, which predicts that the active-site His residue initiates the reaction. However, partial activity in the absence of the His residue is compatible with the direct-attack mechanism, because solvent protons could support turnover at a reduced rate. This catalytic defect should also be ameliorated at high enzyme-to-substrate ratios such as those that prevail in our CdiC overexpression experiments.

TAATs modify specific Lys residues within cognate protoxins, though the recognition determinants remain poorly understood (32). Acylated residues Lys564 and Lys690 of HlyA are found in Gly-Lys motifs, but the surrounding sequences are otherwise unrelated (67). Moreover, only one of the corresponding Lys residues in CyaA is acylated under physiological conditions (68). The sequence context of the modified Lys1467 residue in CdiA^STEC4^ is also unrelated to the acylated segments of HlyA and CyaA. Given that we only examined the RBD region for acylation, it remains possible that other sites within CdiA^STEC4^ (or CdiB^STEC4^) are lipidated by CdiC. However, any additional modifications have little functional significance under laboratory conditions, because the Lys1467Arg substitution in CdiA^STEC4^ recapitulates the Δ*cdiC* phenotype. Inspection of class IV systems from different bacteria also suggests that Lys1467 is the primary modification site. Although most systems encode acyltransferases with TAAT catalytic motifs (Fig. S7), at least four loci lack functional *cdiC* genes. Salmonella enterica strain S-1643 carries a frameshift mutation in *cdiC*, and the clusters from *Methylomusa*, *Sporomusaceae*, and *Rhodoferax* lack *cdiC* altogether. Strikingly, CdiA proteins from these latter systems have substitutions at Lys1467, but this position is always a Lys residue in effectors from *cdiC^+^* gene clusters (Fig. S5 and S6). For S. enterica S-1643, the selective pressure to retain a modifiable Lys residue may be relieved because the strain also harbors a *cdiB* mutation that should preclude effector export. In contrast, *Methylomusa*, *Sporomusaceae*, and *Rhodoferax* probably produce functional effectors, because unmodified CdiA^STEC4^ retains significant growth inhibition activity. Presumably, class IV systems first evolved to recognize LPS in the absence of posttranslational modification and then later acquired a lysyl acyltransferase that augments target cell binding. These *cdiC*-less gene clusters could thus be representative of the ancestral class IV system.

10.1128/mBio.02530-21.7 FIG S7 Alignment of predicted CdiC lysyl acyltransferases. Predicted CdiC proteins from the bacterial strains listed in Fig. S6 were aligned with Clustal Omega and the results rendered using Jalview. Sequences are from the following bacterial strains (NCBI reference identifiers in parentheses), E. coli STEC_O31 (WP_001243916.1), Klebsiella sp. strain RIT-PI-d (WP_049840268.1), Pseudomonas sp. strain RIT357 (WP_032887925.1), Pseudomonas fluorescens E24 (WP_078827813.1), Ralstonia solanacearum GMI100 (WP_011004363.1), Pseudomonas *gingeri* F1001 (WP_177143638.1), Herbaspirillum huttiense NFYY 53159 (WP_134221495.1), *Roseateles* sp. strain YR242 (WP_092949727.1), *Orbus* sp. strain IPMB12 (WP_166917330.1), *Trinickia symbiotica* JPY-366 (WP_107149767.1), and Vogesella mureinivorans 389 (WP_147695852.1). The catalytic dyads corresponding to His37 and Asp107 of CdiA^STEC4^ are marked by red stars and rendered in red font. Download FIG S7, PDF file, 0.9 MB. Copyright © 2021 Halvorsen et al. 2021 Halvorsen et al. https://creativecommons.org/licenses/by/4.0/ This content is distributed under the terms of the Creative Commons Attribution 4.0 International license.

Mass spectrometry suggests that CdiA^STEC4^ is acylated with 3-hydroxydecanoate, which appears to be a novel lipid substrate for a TAAT. HlyC and CyaC were initially reported to be specific for tetradecanoyl and hexadecanoyl groups (respectively) (67, 68), but later studies found that they also append a mixture of odd-length and hydroxylated fatty acids (69, 70). Given that class IV CdiA probably binds to the LPS core directly, we propose that the 3-hydroxydecanoyl moiety enters the hydrophobic leaflet to anchor the interaction. This amide-linked lipid may even mimic the *N-*linked 3-hydroxytetradecanoyl chains of lipid A. It is also notable that the sequence surrounding Lys1467 is basic and contains several Tyr residues (Fig. 7A). The electropositive side chains could interact not only with HepI-phosphate but also with the phosphorylated glucosamine residues that comprise the lipid A backbone. The Tyr cluster could be positioned at the interface between aqueous solvent and hydrophobic bilayer, similar to the circumferential belt of aromatic residues that occupy this zone in all transmembrane β-barrel proteins (71). These biochemical features are strikingly similar to those of polymyxin antibiotics, which are amphiphilic cyclic peptides that carry short, amide-linked aliphatic groups (72). Polymyxins bind initially to the anionic LPS core through cationic diaminobutyric acid residues and then insert their hydrophobic alkyl chains into the bilayer to disrupt outer membrane integrity (73). These parallels strongly suggest that lipidated CdiA effectors utilize the same biophysical strategy to bind Gram-negative target bacteria.

## MATERIALS AND METHODS

### Bacterial strains.

Bacterial strains and plasmids are listed in Table S1. All bacterial cells were grown at 37°C in lysogeny broth (LB) or on LB agar. Where appropriate, media were supplemented with antibiotics at the following concentrations: ampicillin (Amp), 150 μg/ml; chloramphenicol (Cm), 33 μg/ml; kanamycin (Kan), 50 μg/ml; gentamicin (Gm), 15 μg/ml, spectinomycin (Spm), 100 μg/ml; and tetracycline (Tet), 15 μg/ml.

10.1128/mBio.02530-21.8 TABLE S1 Bacterial strains and plasmids used in this studyTable S1, DOCX file, 0.07 MB. Copyright © 2021 Halvorsen et al. 2021 Halvorsen et al. https://creativecommons.org/licenses/by/4.0/ This content is distributed under the terms of the Creative Commons Attribution 4.0 International license.

The *waaC*, *waaP* and *waaY* genes were deleted by phage λ Red-mediated recombineering as described previously (74, 75). Upstream and downstream homology fragments were amplified from E. coli MG1655 using primer pairs CH4195/CH4196 and CH4197/CH4198 (*waaC*), CH4199/CH4200 and CH4201/CH4202 (*waaP*), and CH4203/CH4204 and CH4205/CH4206 (*waaY*) (oligonucleotide primers are listed in Table S2). Upstream and downstream homology fragments were sequentially ligated to plasmid pKAN using SacI/BamHI and EcoRI/KpnI restriction sites (respectively) to generate pCH13508 (Δ*waaC*), pCH13509 (Δ*waaP*), and pCH13510 (Δ*waaY*). These plasmids were PCR amplified with the appropriate outer primer pairs, treated with DpnI, and then electroporated into E. coli CH7175 cells carrying plasmid pSIM6 (75). Recombinants were selected on Kan-supplemented LB agar. The Δ*waaF*::*kan*, Δ*waaG*::*kan*, and *ΔwaaQ*::*kan* alleles were amplified from the Keio collection (76) with primer pairs CH4299/CH4300, CH5507/CH5508, and CH5509/CH5510 (respectively), and the products were recombineered as described above. The *ΔompC*::*kan*, Δ*tsx*::*kan*, and *bamA*(*ΔL6*) alleles were transferred into E. coli CH7175 carrying plasmid pCH9674 by phage P1-mediated transduction to generate strains CH5775, CH5777, and CH5776, respectively. The arabinose-inducible *cdiC* construct was integrated into the *glmS* locus of E. coli MC1061 using Tn*7*-mediated transposition. Triparental mating was performed with MC1061 recipients and MFD donor strains that carry pTNS2 and pCH4872 for 4 h at 37°C. Integrants were selected on Gm-supplemented LB agar, and the insertions were verified by colony PCR using primers CH4672/CH4616. The same mating procedure was used to generate strains CH15163 and CH15164, which carry gentamicin and kanamycin resistance cassettes (respectively) at the *glmS* locus.

10.1128/mBio.02530-21.9 TABLE S2 Oligonucleotides used in this studyTable S2, DOCX file, 0.05 MB. Copyright © 2021 Halvorsen et al. 2021 Halvorsen et al. https://creativecommons.org/licenses/by/4.0/ This content is distributed under the terms of the Creative Commons Attribution 4.0 International license.

### Plasmid constructions.

The *cdiBCAI* gene cluster (*cdiB*, ECSTECO31_0849; *cdiC*, ECSTECO31_0850; *cdiA*, ECSTECO31_0851; *cdiI* is not annotated) was amplified from Escherichia coli STEC_O31 (taxid: 754081) genomic DNA using primer pair ZR258/ZR259 and ligated to pET21b via NotI/XhoI restriction sites to generate plasmid pCH13167. We note that as annotated, *cdiB* does not encode a predicted signal peptide, suggesting that translation actually initiates from a UUG codon 18 nucleotides (nt) upstream. To facilitate further manipulation with restriction enzymes, the *cdiBCAI*^STEC4^ cluster was subcloned into pCH13658 (19) using NotI/XhoI, and a silent XbaI site was introduced at Ser2159 using primers CH4803/CH4804 to generate plasmid pCH1055. The *cdiB*^STEC4^ gene was amplified with CH5654/CH4964 and the product was ligated to pCH1055 via HindIII/NcoI to generate plasmid pCH1145, in which the predicted P*_cdi_* promoter is deleted. The Cys1243Ser mutation was introduced with primers CH4860/CH4803 and the fragment was ligated to pCH1055 using EcoRI/XbaI to generate plasmid pCH1138. Point mutations were introduced into the receptor-binding domain coding sequence using the megaprimer PCR method (77). The Lys1467Ala and Lys1469Ala substitutions were made by PCR using CH4647/CH4803 and CH4648/CH4803. The resulting products were used as megaprimers with CH4802 to generate the final products, which were ligated to pCH1055 via EcoRI/XbaI, yielding plasmids pCH1140 and pCH1058 (respectively). Lys1466Ala, Lys1467Arg, and Lys1467Gln megaprimers were made using CH4802/CH4841, CH4802/CH4888, and CH4802/CH5186 and then paired with primer CH4803 to produce fragments that were ligated to pCH1055 via EcoRI/XbaI to generate plasmids pCH1139, pCH4472, and pCH6884 (respectively). Coding sequences for the minimal RBD^STEC4^ (Val1328 to Pro1589) variants were amplified with CH4358/CH4359 and ligated to pACYCDuet using NcoI/XhoI restriction sites to generate plasmids pCH14508 (wild type), pCH14660 (Lys1466Ala), pCH14661 (Lys1467Ala), pCH14662 (Lys1469Ala), pCH15099 (Lys1467Arg), and pCH7391 (Lys1467Gln). For the cell surface binding assay, the wild-type RBD^STEC4^ coding sequence together an FHA-1 repeat (residues Val1269 to Pro1589) was amplified with CH4991/CH4359 and ligated to pET21b using NheI/XhoI to generate plasmid pCH15160. The RBD^STEC4^ coding fragment was also subcloned into pACYCDuet using NdeI/XhoI to generate plasmid pCH15268. The *cdiI*^STEC4^ immunity gene was amplified with primers CH4869/ZR259 and ligated to pCH405Δ using KpnI and XhoI sites to generate plasmid pCH1061.

To inactivate *cdiC*, a PCR fragment generated with primers ZR258/ZR253 was digested with AscI/NotI and ligated to pCH1055 to produce pCH4469. The resulting construct contains an in-frame deletion of *cdiC* codons corresponding to Ser10 through Glu158. His37Ala and Asp107Ala missense mutations were introduced into *cdiC* using overlap extension PCR (OE-PCR) (78). Fragments generated with primer pairs ZR258/CH4177 and CH4176/CH4088 (for His37Ala) and ZR258/CH4175 and CH4174/CH4088 (for Asp107) were combined using OE-PCR and then ligated into pCH1055 using NotI and AscI restriction sites to generate plasmids pCH4470 (His37Ala) and pCH4471 (Asp107Ala). Wild-type and mutant alleles of *cdiC* were amplified using primers CH4087/CH4088 and ligated to pTrc99KX via KpnI/XhoI to generate plasmids pCH6962 (wild type), pCH14181 (His37Ala), and pCH14182 (Asp107Ala) for *in vivo* lipidation experiments. The wild-type *cdiC* KpnI/XhoI fragment was also ligated to pCH450KX to generate plasmid pCH296, which places the gene under the control of an arabinose-inducible promoter. An NsiI/XhoI fragment containing *araC*, the P_BAD_ promoter, and *cdiC* was excised from pCH296 and subcloned into pUC18R6k-miniTn7T-Gmr to generate the pCH4872 vector for Tn*7*-mediated integration of *cdiC* at the *glmS* locus for complementation experiments. For plasmid-based complementation, *waaC* (CH4387/CH4388), *waaF* (CH4207/CH4208), and *waaP* (CH4209/CH4210) fragments were PCR amplified from E. coli MG1655 and ligated to pCH450KX using KpnI and XhoI restriction sites to generate pCH14473, pCH13581, and pCH13582 (respectively). The E. coli *recA* gene was amplified from MG1655 genomic DNA using primers CH2131/CH2132 and ligated to pSIM6 (75) via BglII/XmaI restriction sites to generate plasmid pCH9674, in which the phage λ *gam*, *beta*, and *exo* recombinase genes are replaced with *recA*.

### Competition cocultures.

All target cell strains were derivatives of CH7175 (E. coli EPI100 Δ*wzb*), and inhibitor strains were derivatives of either E. coli MC1061 or E. coli MG1655. Prior to coculture, inhibitor and target cells were grown separately to an optical density at 600 nm (OD_600_) of 0.6 to 0.9 in LB medium at 37°C. For liquid medium competitions, inhibitors and targets were seeded at a 1:1 ratio (OD_600_ = 0.3) in 10 ml of prewarmed LB medium and then incubated in a baffled flask with shaking at 220 rpm for 3 h at 37°C. For solid medium competitions, mid-log-phase cells were collected by centrifugation, adjusted to an OD_600_ of 3.0, and then mixed at a 1:1 ratio for spotting (15 μl) onto LB agar. After 3 h at 37°C, cells were harvested with a sterile swab into 1× M9 salts. Cocultures were subjected to serial dilution in 1× M9 salts and plated onto antibiotic-selective LB agar to enumerate viable inhibitor and target cells as CFU. Competitive indices were calculated as the ratio of inhibitor to target cells at 3 h divided by the initial ratio. Reported data are the averages ± standard errors for at least three independent experiments.

### Transposon mutagenesis.

MFD *pir^+^* cells carrying plasmid pSC189 were used as donor cells to introduce the *mariner* transposon into E. coli CH7175 cells by conjugation (79). Donors and recipients were grown to mid-log phase in LB medium supplemented with 30 μM diaminopimelic acid and then mixed and plated onto LB agar at 37°C for 5 h. Cells from six independent matings were harvested separately and plated onto Kan-supplemented LB agar to select for transposon mutants. Each transposon mutant pool was harvested into 1 ml of 1× M9 salts and cocultured with E. coli MC1061 carrying pCH13167 to select for CDI^R^ clones. Surviving target bacteria were recovered from the competition cocultures on Kan-supplemented LB agar and subjected to two additional cycles of CDI^R^ selection. CDI^R^ clones were picked randomly from each independent mutant pool, and chromosomal DNA was isolated to identify transposon insertion sites. DNA was digested with NspI overnight at 37°C, followed by enzyme inactivation at 65°C for 20 min. The digests were then supplemented with 1 mM ATP and T4 DNA ligase and incubated overnight at 16°C. The reactions were electroporated into E. coli DH5α *pir*^+^ cells, and transformants were selected on Kan-supplemented LB agar. The isolated plasmids were sequenced using oligonucleotide CH2260 to identify the junctions between the *mariner* transposon and genomic DNA.

### LPS extraction and analysis.

Overnight cultures of E. coli were adjusted to an OD_600_ of 2.0 in 2.0 ml of LB medium and LPS was isolated using an LPS extraction kit (iNtRON Biotechnology). Purified LPS (∼9 μg) was resolved on 13% polyacrylamide SDS gels for 1 h at 110 V. Gels were stained with Pro-Q Emerald 300 lipopolysaccharide gel stain (Thermo Fisher) and imaged on a Kodak 200 Gel Logic UV transilluminator. The LPS standard from E. coli serotype O55:B5 was provided by the kit.

### Cell binding assay.

E. coli cells (derivatives of EPI100 Δ*wzb*) were adjusted to an OD_600_ of 1.0 in 0.5 ml of LB medium and incubated with purified RBD^STEC4^-His_6_ (from pCH15160 at a 1 μM final concentration) for 10 min at ambient temperature. Cells were pelleted in a microcentrifuge at 21,000 × *g* for 1.5 min. Supernatant fractions were collected, and the cell pellets were resuspended in 1.0 ml of 50 mM sodium phosphate (pH 6.5). The washed cells were recollected by centrifugation and the pellets frozen at −80°C. Proteins were extracted with 70 μl of urea lysis buffer (8 M urea, 150 mM NaCl, 20 mM Tris-HCl [pH 8.0]) by rapid thawing in a 42°C water bath coupled with vortexing. The supernatant and urea-solubilized fractions were resolved on 12% polyacrylamide gels buffered with Tris-Tricine. Gels were electrotransferred to a polyvinylidene difluoride (PVDF) membrane for anti-His_6_ (Cell Signaling) immunoblot analysis. The membrane was incubated with IRDye 800CW-conjugated goat anti-rabbit secondary antibodies (LI-COR) and visualized on an Odyssey infrared imager.

To produce processed RBD^STEC4^ for solubility testing, unlipidated and lipidated RBD^STEC4^-His_6_ proteins (14 μM final concentration) were incubated with E. coli CH7176 cells (OD_600_ = 4.0) at ambient temperature for 30 min. Proteins were extracted from the cells using 8 M urea, 50 mM sodium phosphate (pH 7.0), and RBD^STEC4^-His_6_ isolated by Ni^2+^ affinity chromatography. Purified proteins were exchanged into 50 mM sodium phosphate (pH 6.5) on a PD Miditrap G-25 column (GE Healthcare) and concentrated to >5 μM in a 10-kDa Amicon Ultra 0.5-ml centrifugal filter (Millipore). Lipidated and unlipidated proteins were centrifuged at 199,000 × *g* for 5 min in a Beckman Airfuge, and the supernatants were transferred to new tubes. Note that no precipitate was visible. The centrifuge tubes were then washed with 50 mM sodium phosphate (pH 6.5) and centrifuged again for 2 min. The pellet and supernatant fractions were resolved by SDS-PAGE, and the gels were stained with Coomassie brilliant blue.

### Fluorescent dye labeling and immunoblot analysis.

Cells expressing plasmid-borne *cdiBCAI*^STEC4^ gene clusters were grown in LB to an OD_600_ of ∼1.0 at 37°C. Cells were collected from 1.0 to 2.0 ml of culture by centrifugation and washed twice with 1× phosphate-buffered saline (PBS) supplemented with 10 mM MgSO_4_. Washed cells were resuspended in 100 μl of 1× PBS, 10 mM MgSO_4_ with maleimide-IRDye680 (LI-COR) and incubated in the dark for 15 min. Labeling reactions were quenched with 15 mM β-mercaptoethanol, and the cells were washed once with 15 mM β-mercaptoethanol in 1× PBS and 10 mM MgSO_4_. Cells were collected by centrifugation and frozen at −80°C. Frozen cell pellets were resuspended in 50 to 80 μl of urea lysis buffer (8 M urea, 150 mM NaCl, 20 mM Tris-HCl [pH 8.0]) and refrozen at −80°C. Cells were broken by rapid thaw in a 42°C water bath coupled with vigorous vortexing. Urea-soluble protein extracts were quantified by the Bradford method, and equivalent protein loads were resolved by SDS-PAGE on either 6% or 6%/10% polyacrylamide gels buffered with Tris-Tricine. Gels were imaged on an Odyssey infrared imager (LI-COR) and then stained with Coomassie brilliant blue R-250. Dye-labeled and Coomassie-stained proteins were quantified using Odyssey v3.0 and Image Studio Lite v5.2 software packages from LI-COR.

Protein samples for immunoblot analyses were extracted from E. coli cells by freeze-thaw cycles in urea lysis buffer as described above. Urea-soluble proteins were run on 10% polyacrylamide gels at 100 V for 2 h and then electroblotted to PVDF membranes for 1 h at 17 V. Membranes were incubated with rabbit polyclonal anti-OmpC (MyBioSource, San Diego, CA) or rabbit polyclonal anti-BamA (a gift from Thomas Silhavy, Princeton University) antibodies. After washing, the membranes were incubated with IRDye 800CW-conjugated goat anti-rabbit secondary antibodies (LI-COR) and visualized on an Odyssey infrared imager.

### Protein purification and RP-HPLC analyses.

For *in vivo* lipidation assays, E. coli CH2016 cells carrying CdiC and RBD^STEC4^ expression plasmids were grown at 37°C in 150 ml of Cm- and Amp-supplemented LB medium. Once the cultures reached an OD_600_ of ∼2.5, isopropyl-β-d-thiogalactoside (IPTG) was added to a final concentration of 1 mM and the cultures were incubated for 1 h. Cells were harvested by centrifugation and broken by one freeze-thaw cycle (at −80°C) in 12 ml of 6 M guanidine-HCl and 10 mM Tris-HCl (pH 8.0). Unbroken cells and debris were removed by centrifugation at 15,000 rpm in an SS-34 rotor for 15 min. Clarified lysates were adjusted to 10 mM imidazole, and 300 μl of Ni^2+^-nitrilotriacetic acid (NTA) agarose resin was added to bind His_6_-tagged RBD^STEC4^. Resins were batch washed three times with 8 M urea and 10 mM imidazole and twice with 8 M urea. RBD^STEC4^ variants were then eluted with 8 M urea and 200 mM acetic acid. Protein concentrations were determined using an extinction coefficient at 280 nm of 20,400 M^−1 ^cm^−1^ (Val1269 to Pro1589) and 17,420 M^−1 ^cm^−1^ (Val1328 to Pro1589). Circular-dichroism spectroscopy was performed with 5 μM RBD^STEC4^ in 20 mM sodium phosphate (pH 6.5) using a 0.1-cm-path-length quartz cuvette. Purified RBD^STEC4^ proteins were analyzed by reverse-phase high-performance liquid chromatography (RP-HPLC) using a Waters 1525 binary pump controlled by Breeze2 software. Samples were passed through a 0.22-μm cellulose acetate spin filter (Costar) and then injected onto a Vydac 15- by 300-mm C4 column in buffer A (0.06% trifluoroacetic acid) at a flow rate of 1 ml/min. After 5 min, the column was developed with a 0 to 100% linear gradient of buffer B (0.052% trifluoroacetic acid in 80% acetonitrile) over 60 min, and eluted proteins were detected by absorbance at 214 nm using a Waters UV spectrophotometer. HPLC-purified RBD^STEC4^ proteins were dried by SpeedVac and redissolved in formic acid for electrospray ionization-mass spectrometry. Dried HPLC-purified samples were also dissolved in 8 M urea for peptide mapping. Lipidated and unmodified RBD^STEC4^ (1 nmol) was digested in 2 M urea, 50 mM Tris-HCl (pH 7.5), 2.5 mM CaCl_2_, and 5 mM dithiothreitol with 50 μg/ml of endoproteinase Arg-CT (Worthington Biochemical) at 37°C for 2 h. Digests were injected onto a Vydac 15- by 300-mm C4 column in buffer A at 1 ml/min, and peptides were eluted with a linear gradient of 0 to 100% buffer B over 60 min.
