# Supplementary material for: Lipidation of Class IV CdiA Effector Proteins Promotes Target Cell Recognition during Contact-Dependent Growth Inhibition
Source: mBio. 2021 Oct 12;12(5):e02530-21. doi: 10.1128/mBio.02530-21 (PMC8510554; doi:10.1128/mBio.02530-21)
Supplement: FIG S1 [file mbio.02530-21-sf001.pdf]

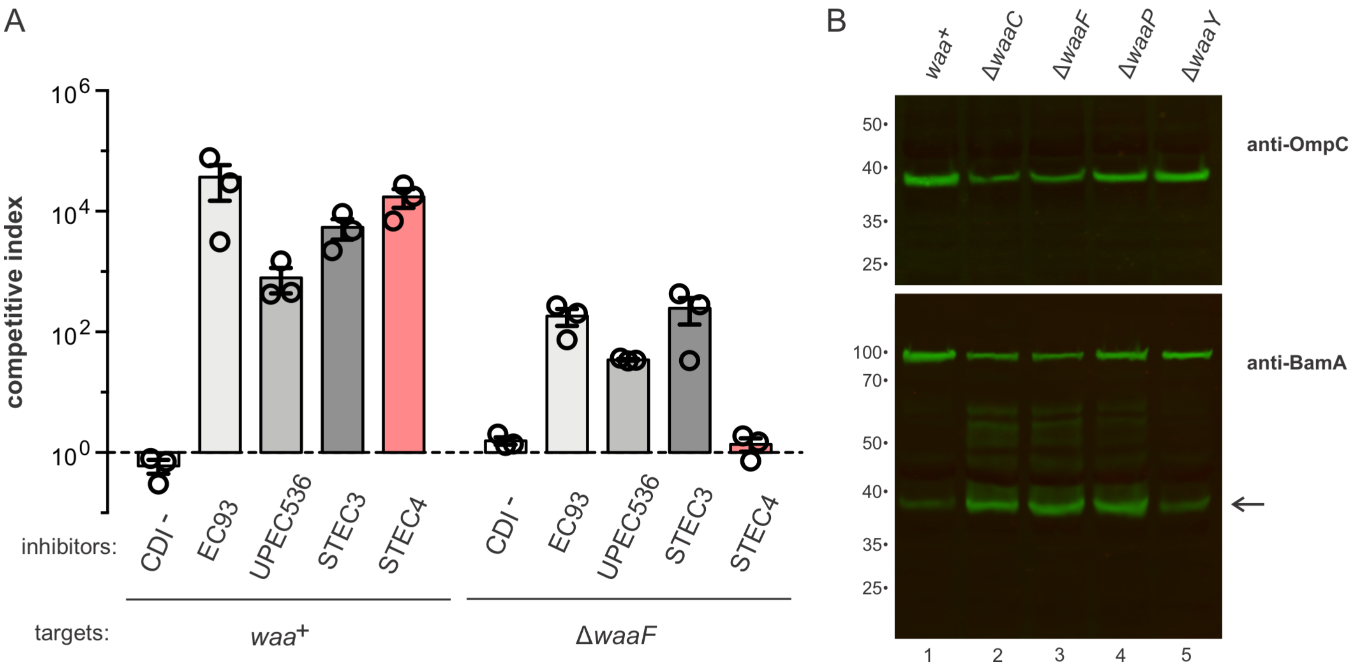

Figure S1. ***E. coli*** **deep-rough mutants are partially resistant to OMP targeting CdiA proteins. A**) Inhibitor strains expressing the indicated CDI systems were co-cultured at a 1:1 ratio with *E. coli* CH7175 (*waa*+) or CH13816 (Δ*waaF*) target cells on LB agar. The competitive index is the ratio of viable inhibitor to target cells after 3 h. Data are the average ± SEM from three independent experiments. **B**) Total urea-soluble protein was isolated from the indicated *waa* backgrounds for immunoblot analysis using polyclonal antibodies to OmpC and BamA. The arrow indicates an apparent BamA degradation product that accumulates in deep-rough mutant backgrounds.
