# Supplementary material for: Lipidation of Class IV CdiA Effector Proteins Promotes Target Cell Recognition during Contact-Dependent Growth Inhibition
Source: mBio. 2021 Oct 12;12(5):e02530-21. doi: 10.1128/mBio.02530-21 (PMC8510554; doi:10.1128/mBio.02530-21)
Supplement: FIG S2 [file mbio.02530-21-sf002.pdf]

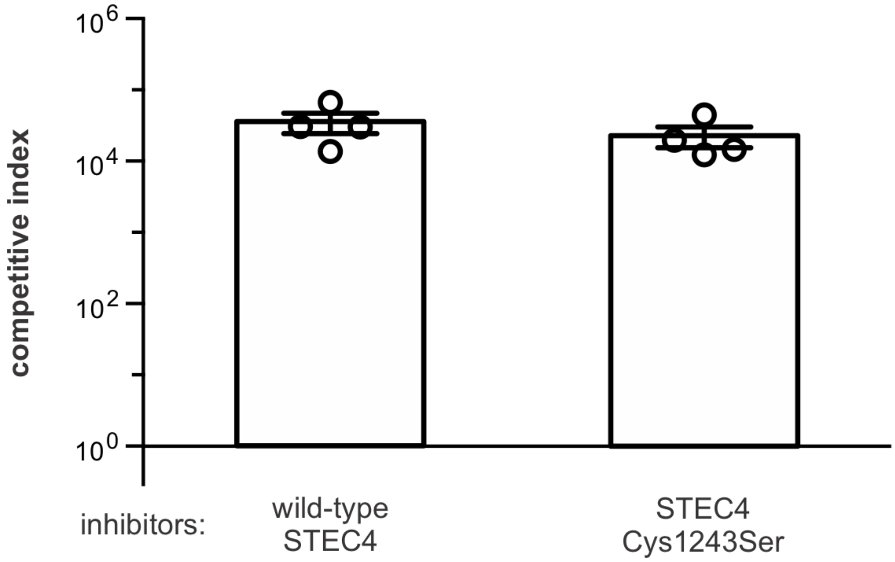

Figure S2. The Cys1243Ser substitution has no effect CdiA^{STEC4} growth inhibition activity. Inhibitor cells expressing wild-type CdiA^{STEC4} or the Cys1243Ser variant were co-cultured at a 1:1 ratio with *E. coli* CH7175 (*waa*$^+$ Δ*wzb*) target cells on LB agar. The competitive index is the ratio of viable inhibitor to target cells after 3 h. Data are the average ± SEM from four independent experiments.
