# Supplementary material for: Lipidation of Class IV CdiA Effector Proteins Promotes Target Cell Recognition during Contact-Dependent Growth Inhibition
Source: mBio. 2021 Oct 12;12(5):e02530-21. doi: 10.1128/mBio.02530-21 (PMC8510554; doi:10.1128/mBio.02530-21)
Supplement: FIG S3 [file mbio.02530-21-sf003.pdf]

```
EC93        ---------------------------------------------MHQPPVRFTYRLLSY 15
STECO31_IV  MNRNCYRIIFNKARGMLMVVADIARSGRAGTSLSSRTGYPHRQRICRVTPLAFSLWLASG 60
                                                          :  *: *:   * *

EC93        LVSAIIAGQPLLPAVGAVITPQNGAGMDKAANGVPVVNIATPNGAGISHNRFTDYNVGKE 75
STECO31_IV  MVHSVNAAG-IAADHG--APGHQQPTITQTASGIPQVNIQTPSAGGVSHNTYSQFDVGNK 117
            :* :: *.  :    *     ::   : ::*.*:* *** **...*:*** :::::**::

EC93        GLILNNATGKLNPTQLGGLIQNNPNLKAGGEAKGIINEVTGGKRSLLQGYTEVAGKAANV 135
STECO31_IV  GVILNNAHNNV-QTQLGGMVAGNPWLA-KGEARIILNEVNSRNPSQLNGFVEVAGKKAQV 175
            *:***** .::  *****:: .** *   ***: *:***.. : * *:*:.***** *:*

EC93        MVANPYGITCDGCGFINTPHATLTTGKPVMNADGSLQALEVTEGSITINGAGLDGTRSDA 195
STECO31_IV  VIANPAGISCDGCGFINANRATLTTGQPQMK-NGSLTGFSVERGEIQITGKGMDASRTDY 234
            ::*** **:********: :******:* *: :*** .:.* .*.* *.* *:*.:*:*

EC93        VSIIARATEVNAALHAKDLTVTAGANRITADGRVS--ALKGEGNVPKVAVDTGALGGMYA 253
STECO31_IV  TDIIARSVKINAGIWAQDLKVTTGRNNVDIAHGQTEKKAADASSQPQVALDVSSLGGMYA 294
            ..****:.::**.: *:**.**:* *.:      :     . .. *:**:*..:******

EC93        RRIHLTSTESGVGVNLG-NLYAREGDIILSSSGKLVLKNSLAGGNTTVTG--TDVSLSGD 310
STECO31_IV  GKIRLVGTETGVGVRNAGHIGAQAGAVTLTADGRIENSGSISAKTDVHLATTRELHNSGS 354
             :*:*..**:****. . :: *: * : *::.*::  ..*::. . .  .   ::  **.

EC93        NKAGGNLSVTGTTGLTLNQSRLVTDKNLVLSSSGQIVQNGGELTAGQNAMLSAQHLNQTS 370
STECO31_IV  VYAGQDTQIQS-NGVFTHTGSVASRRNTR--------IQTARLTGGERSLLAAGV--KDD 403
              ** : .: . .*:  : . :.: :*           : ..**.*:.::*:*    : .

EC93        GTVNAAENVTLTTTDDTTLKGRSVAGKTLTVSSGSLNNGGTLVAGRDATVKTGTFSNTGT 430
STECO31_IV  GRLAAAGNLTVSTTGELAAHGQ-------------------VLSGGDMQL---------- 434
            * : ** *:*::**.: : :*:                   :::* *  :

EC93        VQGNGLKVTATDLTSTGSIKSGSTLDISARNATLSGDAGAKDRALVTVSGTLENRGRLVS 490
STECO31_IV  -KGQGLDLSNSRIQGQHTELDATSGNLSTQNVQLSAG-----TLSARTAGHFSNNGGTIN 488
             :*:**.:: : : .  :  ..:: ::*::*. **..        . .:* :.*.*  :.

EC93        DDVLTLSATQINNSG-TLSGAKELVASADTLTTTEKSVTNSD------GNLMLDSASSTL 543
STECO31_IV  ADILQISAQSLSNHRGKLIQT----GTGDFSLNLPGGVDNREGLLAANGAVRLDALSL-- 542
             *:* :** .:.*   .*  :    .:.*   .   .* * :      * : **: *

EC93        AGETSAGGTVSVKGNSLKTTTTAQ--TQGN------SVSVDVQ-----NAQL-------- 582
STECO31_IV  --DNRRGKVQAVQSGSLQVKTTGAVDNQQGSLTASRDVRLNAQALNNDNGLISAAAGTGR 600
              :.  * . :*:..**:..**.   .* .      .* ::.*     *. :

EC93        ----------DGTQAARDILTLNASEKLTHSGKSSAPSLSLS--APELTSSGVLVG-SAL 629
STECO31_IV  IKTQQAVSNTEGRMESAGRLGISAGSLNNHQGTVVSDGLSVTLDGALDNTSGRLLSQKTL 660
                      :*   : . * :.*..  .*.*.  : .**::  .   .:** *:. .:*

EC93        N-TQSQTLTNSGLLQGKASLTVNTQR--LDN---QQNGTLYSAADLTLDIPDI-RNSGLI 682
STECO31_IV  SVSGSELVSDDGLIQSGSDMTLDVQDGVLSNRNTKTRGGISSAGTLTVRAGMLNNQQGFI 720
            . : *: :::.**:*. :.:*::.*   *.*   : .* : **. **:    : .:.*:*

EC93        TGDNGLMLNAVSLSNPGKIIADTLSVRATTLDGDGLLQGAGALALAGDTLSLGSNGRWLT 742
STECO31_IV  VGQKDMTLNAGTLDNRQGVLGSQASLQ---ISSGTLMNQKGALKAGTDM--------LLS 769
            .*::.: *** :*.*   ::..  *::   :... *::  ***  . *          *:
```

```
EC93       AGDLSLRGKTLHTAGTTQGQNLTVQADRWANSGSVQATGNLTASATGQLTSTGDIMSQGD 802
STECO31_IV GGDVSNQEGTL------------------------AAGRDLNAHLNVLENQQGTVVSNGN 805
           .**:* :  **                         *  :*.*  .   .. * ::*:*:

EC93       TTLNAATTDNRGSLLS-AGTLSLDGNSLDNSGTVQGNHVTIRQNGVTNSGTLTGIAALTL 861
STECO31_IV SRLDVTRFDNQGGRLVAQQSLTLSSTDI-----------INDASGLIQSGASLNLRADTL 854
           : *:.:  **:*. *    :*:*....:               .*: :**:  .: * **

EC93       AARMDMASPQPALMNNGGSLLTSGDLTITAGSLANSGAIQA-ADSLTARLTGELVSTAGS 920
STECO31_IV SNR---------NSGDRGGVISQGPMTLNAGTLDSTAGVLLSGDAL--SLTAGVVNNTSG 903
           : *           .: *.:::.* :*:.**:* .:..:   .*:*   **. :*..:..

EC93       KVTSNGEMALSALNLSNSGQWIAKNLTLKANSLTSAGDITGVDALTLTVNQTLNNHASGK 980
STECO31_IV QVVANGLLG-----------WNSQALNNQSG------LIQGRGISINTAGQTLDN-RRGT 945
           :*.:** :.           * :: *. ::.       * * .    *..***:*   *.

EC93       LLSAGVLTLKADSVKNDGQLQGNATTITAGQLTNGGHLQGETLTLAASGGVNNRSGGVLM 1040
STECO31_IV LNSLQELTVSTGAMDNRG-----------------GTVGAKTTADLSTTSLDNREGGRLV 988
           * *   **:.:.::.* *                 * : .:* :  :: .::**.** *:

EC93       SRNALNVSTATLSN-QGTIQGGGGVSLNATDRLQ--NDGKILSGSNLTLTAQ-----VLA 1092
STECO31_IV SEGELRLHTGGLQNSHGQIQSVGDMLLNSVRGVVDNVSGLIRSGSAITLNALQFINRHTQ 1048
           *.. *.: *. *.* :* **. *.: **:.  :    .* * *** :**.*

EC93       NTGSGLVQAATLLLDVVNTVNGGRVLATGSADV-KGTT-LNNTGTFQ-GADLLVN--YHT 1147
STECO31_IV NTGQGLEAQTIHITTQDLDNQEGSILADRALTVMADRTLSNNDGVLSSGATLSVSGRQLA 1108
           ***.**   :  :       : * :**  :  *  . *  ** *.:. ** * *.    :

EC93       FSNSGTLLGTSGLGVKGSSLLQNGTGRLYSAGNLLLD-AQDFSGQGQVVATGDVTLKLIA 1206
STECO31_IV FSNRDGVVK-AGQSVSVDAGQLGGDGKLLSLGNMTLKSNTTFSNSGQTIANGNLTLSVNG 1167
           *** . ::  :* .*. .:   .* *:* * **: *.    **..**.:*.*::**.: .

EC93       ALTNHGTLAAGKTLSVTSQNAVT--NGGVMQGDAMVLGAGEAFTNNGTLTAGKGNSV--- 1261
STECO31_IV DVSNTGSLLAGSRLDLNSIRLENTEKGEISAGQ-TWLNVTDTLLNRGLID-GKYTRLQAN 1225
            ::* *:* **. *.:.* .  .  :* :  *:   *.. ::: *.* :  ** . :

EC93       ----FSAQRLFLNAPGSLQAGGDVSLNSRSDITISGFTGT-AGSLTMNVAGTLL---NSA 1313
STECO31_IV TLTNSGTGRIYGDAVGVCAA-------TFNNLEENGVAATLAGRERVDLGVQTLNNRTHS 1278
                .: *:: :* *   *       : .::  .*.:.* **   :::.   *   . :

EC93       LIYAGNNLKLFTDRLHNQHGDILAGNSLWVQKDSSGT-----ANSEIINRSGNIETTRGD 1368
STECO31_IV LIYS--------------AGDMHTGGMLDANGAATGKAGVLNNHSATIEAAGYLVLSAGQ 1324
           ***:               **: :*. * .:  ::*.      :*  *: :* :  : *:

EC93       ITMNTAHLLNSWDAISASHEVIPGSSHGVISPVPENNRWWGVVR-----HDGVEYLAVYW 1423
STECO31_IV INNVNDHF-------TTERVVVSTEKVTEYQLSGSDKRWSAGEPGVYVDNDSSNSLKKLH 1377
           *.  . *:       ::.: *:  ..    .   .::** .        :*. : *

EC93       -GKGAT-VPDEYRIRTGDTETVTVSASGHAARISGGADMHIRAGRLDNEASFILAGGGMT 1481
STECO31_IV TPEGARDKFTQYDYTR-TVEE-TRVKESDPGKILSGAGMTIVADKLLNDKSQVVAGGLLT 1435
             :**     :*      .*  *   ... .:* .**.* * *.:* *: * ::*** :*

EC93       LSGDTLNNQGWQEGTTGKETVWRLASGSLPKAWFTEPWYKVYRQVSPDAT-EASGTSPAG 1540
STECO31_IV IPSGSVENVSVSG----ERH--------VTDSGTSTYYYRIRKKGKDKQGEKTSQYTPPT 1483
           : ..:::* . .     :.         : .:  :  :*:: :: . .   ::*  :*
```

```
EC93       QYRAVISAAGDVSASFATDTGNTTVMPRAGGAGNTITVPSLNSLTPP--TVSQGVSGEAL 1598
STECO31_IV VIQTITLKPGELTSH-----------GQ--VQGSHVTLS---PLKPQGTDVQTGLTGNVD 1527
             :::    *::::             :    *. :*:     *.*    *. *::*:.

EC93       LNESGTG--ITGPVWNDALPDTLKDIPGALSLSGASVSSYPLPSGNNGYFVPSTDPDSPY 1656
STECO31_IV ATVAGTDRIPLRPVVSAGEP--VILLPGQ------Q-FEVSAPQGSIHVAGPDTR----- 1573
            . :**.     ** . . *  :  :**       .  .   *.*.     *.*

EC93       LITVNPKLDGLGKVDSSLFAGLYDLLRMQPGEAPRETDPAYTDEKQFLGSSYILDRLGLK 1716
STECO31_IV ------------LPDSSLFK----TNPAVNVPYLVETDPRFTNQKTWLGSDYMQKAFSQN 1617
                         *****                ****  :*::* :***.*: . :. :

EC93       PEKDYRFLGDAAFDTRYVSNVILNQTGSRYINGTGSDLAQMKYLMDSAAAQQKALGLTFG 1776
STECO31_IV GDNMLKRLGDGFYEQRLIREQVVALTGQRYLDGYSNDEEQFKALMDAGIAFGKQYNLTPG 1677
            ::  : ***. :: * : : ::  **.**::* ..*  *:* ***:. *  *  .** *

EC93       VSLTAGQVAQLTRSLLWWESVTIN-----GQTVMVPKLYLSPEDITLH-NGSVISGNNVQ 1830
STECO31_IV VALTAEQMALLTGDIVWLVNTTVTLPDGSTQTVQVPQVYARVKPGDVNSAGALIAGRDMV 1737
           *:*** *:* ** .::*  ..*:.      *** **::*   :   ::  *::*:*.::

EC93       -LAGGNITNSGSSINAQNDLLLDRTGSIDNLNAGLINAGGALNLKAIGDIGNISSVISGK 1889
STECO31_IV MKLDGDLFNSGKLAGKQ-----------------------TVQLS-AENIHNQAGTIQGA 1773
              .*:: ***.  . *                       :::*.   :* * :..*.*

EC93       TVSLESATGNISNLTRTEQWAMNNGYNHFSGTDTGPLAAVRATDSLFMGAAGDISITGAA 1949
STECO31_IV NVSL--------------------------------------------TARTDINSTGGL 1789
           .***                                             *  **. **.

EC93       VSAGDSVLLAAGNDLNMNAIQAGERRRYGGSGWYETHAVAP-----TVTAGNSLMLSAGR 2004
STECO31_IV LQATDSLLAMAGRDISLTTTTRTAQRDAGQ-NHFERTRIDSVAGVYVQNDQGRLVLQAGR 1848
           :.* **:*  **.*:.:.:     :*  *  . :*   :       . .  . *:*.***

EC93       DVNSQAAGITAE---NSMDIRAGRDVNMAAESTGAGDHDSTFSM--KTVHDSVRQQGTDM 2059
STECO31_IV DMNLTAATVVNQGKDSLTQLSAGRDMTLSTVTTSAQDNI-TWDKNNRLSQGVTQSTGSTL 1907
           *:*  ** :. :   .  :: ****:.::: :*.* *:  *:.   :  :. .:. *: :

EC93       TSGGDITVTAGRDITSVATAVTAKGDIRVNAGHDIVLGTATES----DYHYSESGETRNR 2115
STECO31_IV AGNGDVTLTAGRDMTSQAASLSAQKGLALMAGHDVTLTGAQNTSSLDEYHKVT---GSSG 1964
           :..**:*:*****:** *::::*: .: : ****:.*  * ::    :**        .

EC93       LLSHQTTRTITEDSVTREKGSLLSGNRVTVNAGNNLTVQGSDVVADRDVSLAADNHVDVL 2175
STECO31_IV MLSKTTTTTHDVSDRRTMTGSELNGDTVSIGAGHNLNVTGSSVAGDNRVSLVAGNNLNIG 2024
           :**: ** *   ..    .** *.*: *::.**:**.* **.*..*. ***.*.*::::

EC93       AATSTDTSWRFKETKTSGLTGTGGIGFTTGSSKTTHDRREAGTTQSQSASTIGSTAGNVS 2235
STECO31_IV MLTESNRETHLKQEKKSGLMSSGGVGFSVGSQSLKV--RDTATDTTQKGSTVGSVHGDVS 2082
             *.:: . ::*: *.*** .:**:**:.**.. .   *::.*  :*..**:**. *:**

EC93       ITAGKQAHISGSDVIANRDISITGDSVVVDPGHDRRTVDEKFEQKKSGLTVALSGAVGSA 2295
STECO31_IV LQAGNRLTVNGSDLIAGRDMALSGKEVSITAATDQHVQTHTVEQKTSGLTLALSGTVGSA 2142
           : **::  :.***:**.**::::*..* :  . *::.  ...***.****:****:****

EC93       INNAVTMAREAKETSDSRLAALKGTQAVLSGVQAGVN---HGLQQQSADPNNGIGVSISL 2352
STECO31_IV LNTTVETVQAAKSAGNSRLEALQGVKAALSGAQAVQAGRLADAQGADAGNNNTVGISLSY 2202
           :*.:*  .: **.:.:*** **:*.:*.***.**       .  *  .*. **  :*:*:*
```

```
EC93       NHQQSKSETKYQHDIVSGSTLSAGNNVSVTATGKNKDHNNSGDMLITGSQIKSGNDTSLN 2412
STECO31_IV GSQSSKSEQQSEQTVAKGSTLTAGNNLSIQATGSGVK-GVDGDLTIQGSQIKAGNNVLLQ 2261
           . *.**** : :: :..****:****:*: ***.. . . .**: * *****:**:. *:

EC93       AQNDILLAAAADTRQTTGKNSSKGGGVGVSFGGGTNGGGLSIFAGINGSEGREKGNGTTW 2472
STECO31_IV ANRDVNLVSAENTSKLEGKNTSSGGSVGVGVGVGSGGWGISVSASANQGKGSEKGNGTTH 2321
           *:.*: *.:* :* :  ***:*.**.***..* *:.* *:*: *. * .:* *******

EC93       TETTLDAGKNVSLTSGRDTTLSGAQVSGEKVTADVGNNLTISSLQDSDRYDSRQNRVAAG 2532
STECO31_IV TETTVDAGNRLTIISGRDTTLTGAQAGGETVKVDAGRHLTLTSEQDSDRYDSKQQNASAG 2381
           ****:***:.::: *******:***..**.*..*.*.:**::* ********:*:..:**

EC93       GSFTFGSMSGSGYASISQDKIKSNYDSVREQSGIYAGKDGFDVTVGNHTQLNGAVIASTA 2592
STECO31_IV GSFTFGSMSGSASVNLSRDKMHSNYDSVQEQTGIFAGRGGFDVTTGQHTQLNGAVIASTA 2441
           ***********. ..:*:**::******:**:**:**:.*****.*:*************

EC93       TDDKNSLNTNTLGWSDIHNQADYKASHTGISLSGGSGMSASQMVASNAIAGAANALTGMS 2652
STECO31_IV TADKNRLDTGTLGFSDIENRADFKTEHQSAGLSTGGSV------AGNFLGNMANNLLVGA 2495
           * *** *:*.***:***.*:**:*:.* . .** *..:      *.* :.. ** *   :

EC93       GSSGHAEGTTSSAISGGNLIIRNKESQKQDIAGLSRDPENANGSIAPIFDREKEQKRLQE 2712
STECO31_IV NHEGHADSTTQSAVSAGNITIRDTKSQKQDVADLNRDAAHANQTLSPIFDREKEHQRLQQ 2555
           . .***:.**.**:*.**: **:.:*****:*.*.**  :** :::********::***:

EC93       AQVISQISGQMSNIVMTYGETEAMKAARKEHPGMSDA---------------QLRETPEY 2757
STECO31_IV AQLIGEIGNQVADIARTEGQIAGEKAKRDPAA-LNQARAELEAAGKPFTEQDVAQRAYNN 2614
           **:*.:*..*:::*. * *:  . ** *.    :.:*                 :.: :

EC93       REVMKGYGTGSTPQMVVQAITGVLGGLNAGNPGQVLAGGLNPAVAQLIKQATGD------ 2811
STECO31_IV GMAASGFGTGGKYQQAIQAATAAVQGLAGGNLSAALAGGAAPYLAEVVKTMTTDPVTGEV 2674
             . .*:***.. * .:** *..: ** .** . .****  * :*:::*  * *

EC93       NREANLMAHAVWGALAAQLGGNNAASGAAGAFSGELAARYIIDNYYGGRTDNLSEQERQQ 2871
STECO31_IV NKAANVTAHAVVNAALAVAQGNNALAGAAGAATGEMVG-MIATQMYGKSVSGLSETEKQT 2733
           *: **: **** .*  *   **** :***** :**:..  *  : **  ...*** *:*

EC93       ISMLATIASGIAGGLVGNSTSAAGTGAQAGRNSVENNAMSGLEGFGTGFQSYVQAQEALV 2931
STECO31_IV LSTLATVAAGLAGGLVGNSGASAVAGAQSGKTTIENNSMSGLVPPRVQQDA------SLA 2787
           :* ***:*:*:******** ::* :***:*:.:::***:****    .  ::      :*.

EC93       NNTNLTDKNGKVLNPATPEEIKYASDKLVTGSIPEGQDPARGLLISWGAGASVFGGELIA 2991
STECO31_IV FDPSQQ---GK-----SAEEISDA-------------IGASHMGPSWGTTYKV---HPIV 2823
            : .     **     : ***. *               *  :  ***:  .*   . *.

EC93       PAVGTVAVIGGTLLGGTTDAVKQFLTLKPGEQYSTTDTLIAAGEGGLTQGKGVIFSTFIN 3051
STECO31_IV QAGGDVSFIRGYTLNGTIDDN--HISVNQGDIYSIGA----HGGASLGLSFGPYFPGLIN 2877
            * * *:.* *  *.** *    .:::: *: **        * ..*  . *  *  :**

EC93       TMGAYLG-SKAKGEDPTGPMVGNAIGTALGNKAGDKFTKEMLSRGFGSVTSEVTGT-VTG 3109
STECO31_IV TNNNDYSINGGFGVGSA--------GITMG-KDGVSFTFG-VGPSWGWSATEIKGVDVNG 2927
           * .   . . . * . :         * ::* * * .**   :. .:*  ::*:.*. *.*

EC93       SVIGTVTDYQIEKLGKGNKEGAK 3132
STECO31_IV TSTNEIYRYDFK----------- 2939
           :  . :  *:::
```

Figure S3. Alignment of class I CdiA^{EC93} and class IV CdiA^{STEC4} effector proteins. The amino acid sequences of CdiA^{EC93} (AAZ57198.1) and CdiA^{STEC4} (WP_001081258.1) were aligned using Clustal Omega at http://www.uniprot.org. Domains and peptide motifs are outlined as determined by the NCBI Conserved Domains sequence analysis site. Pink bold-face indicates the Sec-dependent signal peptide; green indicates the hemagglutinin activity domain (Pfam: PF05860); blue indicates FHA-1 peptide repeats (Pfam: PF05594); orange indicates FHA-2 peptide repeats (PF13332), yellow indicates the pre-toxin-VENN domain (PF04829); and purple indicates the variable CdiA-CT toxins. The receptor-binding domain of CdiA^{EC93} is shown in black bold-face. CdiA^{STEC4} residues Cys1243 and Lys1467 are rendered in red bold font.
