# Supplementary material for: Lipidation of Class IV CdiA Effector Proteins Promotes Target Cell Recognition during Contact-Dependent Growth Inhibition
Source: mBio. 2021 Oct 12;12(5):e02530-21. doi: 10.1128/mBio.02530-21 (PMC8510554; doi:10.1128/mBio.02530-21)
Supplement: FIG S4 [file mbio.02530-21-sf004.pdf]

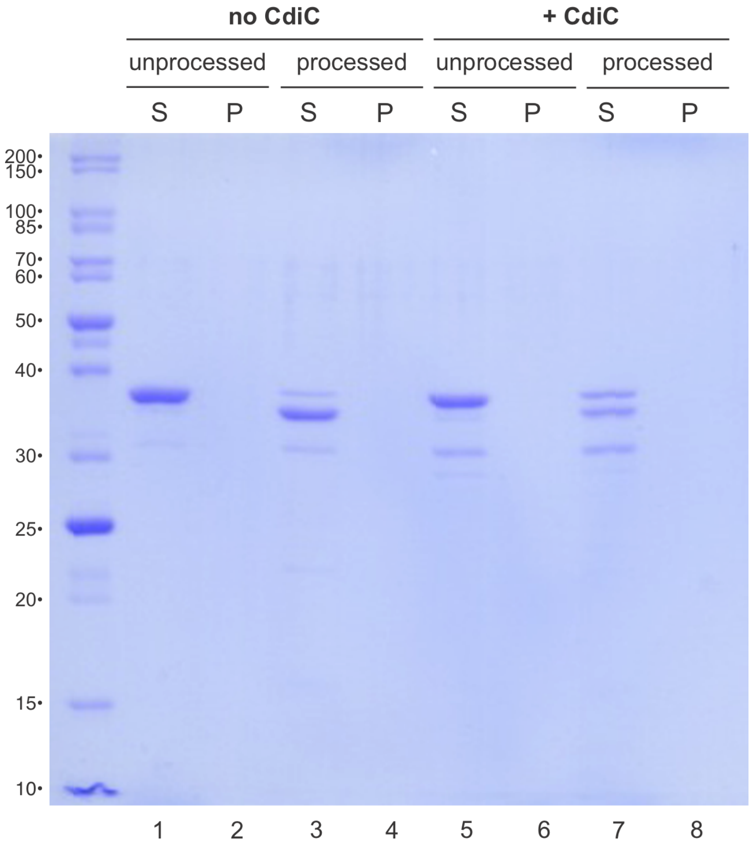

Figure S4. RBD^{STEC4}-His$_6$ remains soluble after cell-dependent N-terminal proteolytic cleavage. Unlipidated (no CdiC) and lipidated (+ CdiC) RBD^{STEC4}-His$_6$ were incubated with *E. coli waa*$^+$ cells to induce N-terminal processing, then the processed proteins were re-isolated by Ni^{2+}-affinity chromatography under denaturing conditions. After buffer exchange into sodium phosphate, processed and unprocessed proteins were centrifuged at 199,000 ×*g* for 5 min into supernatant (S) and precipitate (P) fractions for SDS-PAGE analysis.
