# Supplementary material for: Lipidation of Class IV CdiA Effector Proteins Promotes Target Cell Recognition during Contact-Dependent Growth Inhibition
Source: mBio. 2021 Oct 12;12(5):e02530-21. doi: 10.1128/mBio.02530-21 (PMC8510554; doi:10.1128/mBio.02530-21)
Supplement: FIG S5 [file mbio.02530-21-sf005.pdf]

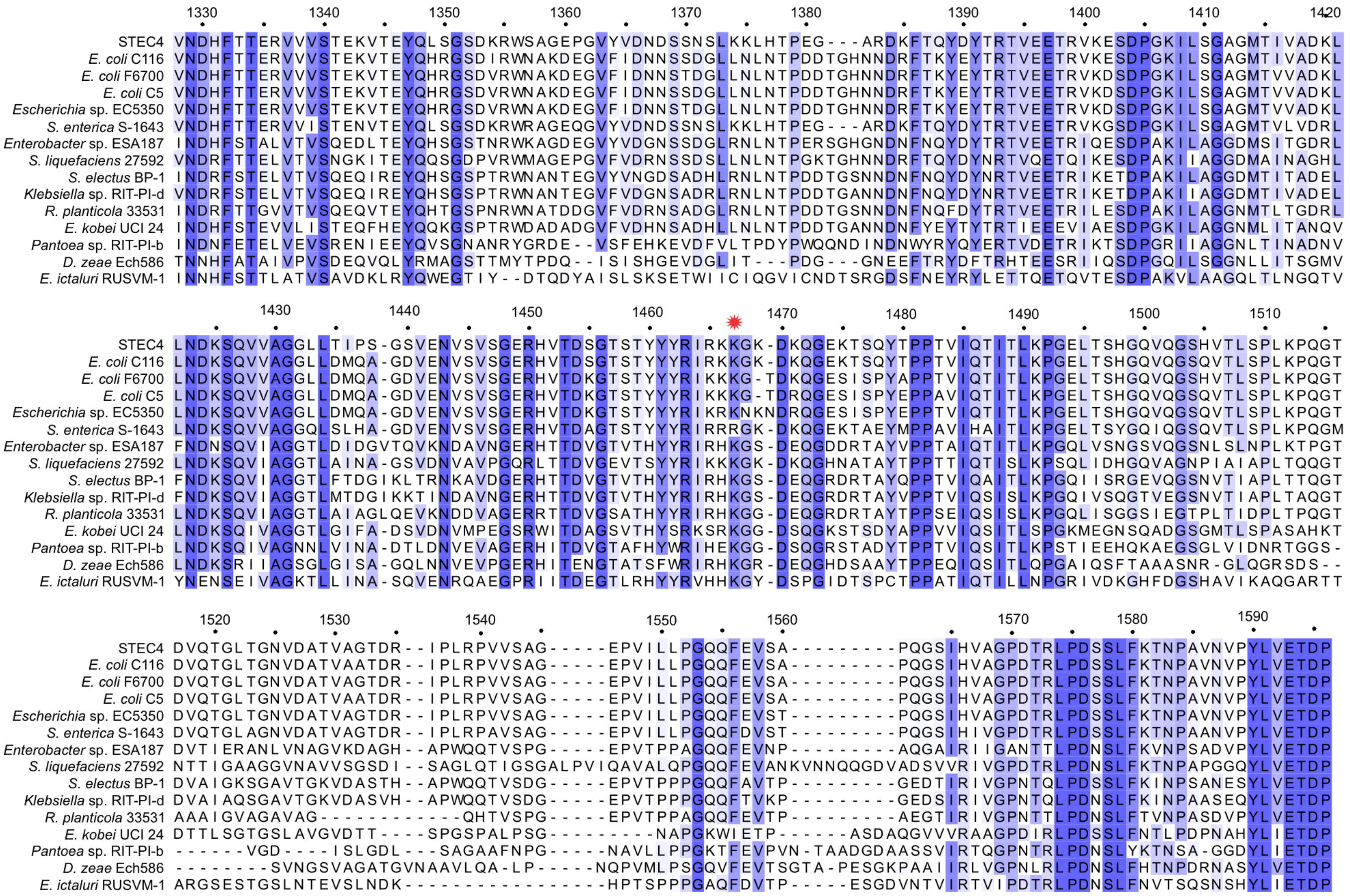

Figure S5. Alignment of enterobacterial class IV RBDs. Predicted class IV effectors were identified by PSI-BLAST searches using CdiA^{STEC4} residues Val1328 – Pro1601 as the query. Hits from enterobacteria were selected for alignment with Clustal omega and the results rendered using Jalview. Protein sequences are from the following bacterial strains (NCBI reference identifiers in parentheses): *E. coli* STEC_O31 (WP_001081258.1); *E. coli* C116 (WP_059337603.1); *E. coli* F6700 (WP_077784734.1); *E. coli* C5 (WP_073521113.1); *Escherichia* sp. MOD1-EC5350 (WP_105289418.1); *Salmonella enterica* serovar Macclesfield str. S-1643 (WP_088731624.1); *Enterobacter* sp. SA187 (WP_083580967.1); *Serratia liquefaciens* ATCC 27592 (WP_020828638.1); *Superficieibacter electus* BP-1 (WP_103750823.1); *Klebsiella* sp. RIT-PI-d (WP_049840269.1); *Raoultella planticola* ATCC 33531 (WP_032700063.1); *Enterobacter kobei* UCI 24 (WP_032668685.1); *Pantoea* sp. RIT-PI-b (WP_049850665.1); *Dickeya zeae* Ech586 (ACZ78808.1); and *Edwardsiella ictaluri* RUSVM-1 (WP_081166522.1). The position corresponding to acylated residue Lys1467 in CdiA^{STEC4} is indicated with a red star.
