# Supplementary material for: Lipidation of Class IV CdiA Effector Proteins Promotes Target Cell Recognition during Contact-Dependent Growth Inhibition
Source: mBio. 2021 Oct 12;12(5):e02530-21. doi: 10.1128/mBio.02530-21 (PMC8510554; doi:10.1128/mBio.02530-21)
Supplement: FIG S6 [file mbio.02530-21-sf006.pdf]

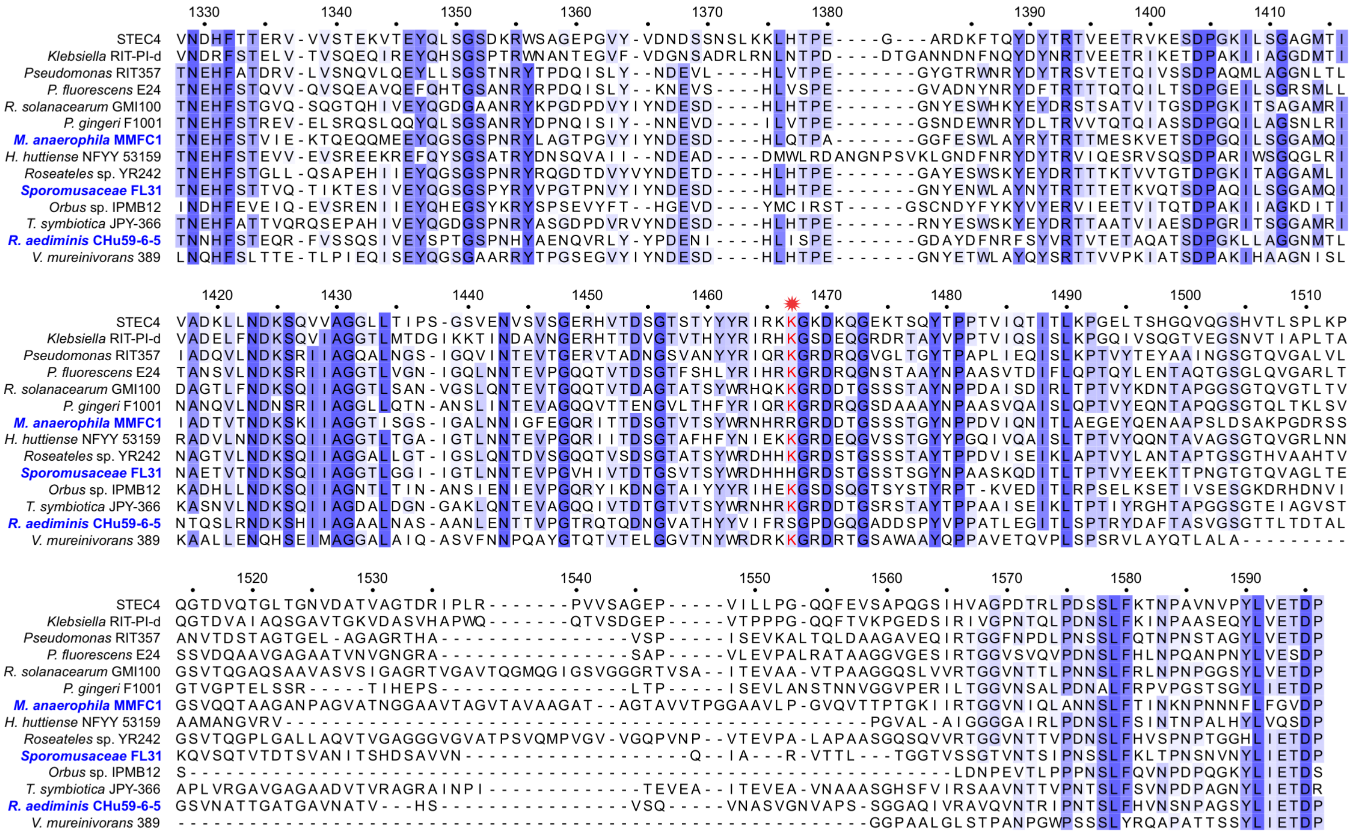

Figure S6. Alignment of predicted class IV RBDs. Predicted class IV effectors were identified by PSI-BLAST searches using CdiA^{STEC4} residues Val1328 – Pro1601 as the query. Hits from diverse phyla were selected for alignment with Clustal omega and the results rendered using Jalview. Protein sequences are from the following bacterial strains (NCBI reference identifiers in parentheses): *E. coli* STEC_O31 (WP_001081258.1); *Klebsiella* sp. RIT-PI-d (WP_049840269.1); *Pseudomonas* sp. RIT357 (WP_032887927.1); *Pseudomonas fluorescens* E24 (WP_078827814.1); *Ralstonia solanacearum* GMI100 (WP_011004362.1); *Pseudomonas gingeri* F1001 (WP_177143639.1); *Methylomusa anaerophila* MMFC1 (WP_126305891.1); *Herbaspirillum huttiense* NFYY 53159 (WP_134221496.1); *Roseateles* sp. YR242 (WP_092949725.1); *Sporomusaceae* bacterium FL31 (GBG57802.1); *Orbus* sp. IPMB12 (WP_166917331.1); *Trinickia symbiotica* JPY-366 (WP_107149766.1); *Rhodoferax sediminis* CHu59-6-5 (WP_142817537.1); and *Vogesella mureinivorans* 389 (WP_147695851.1). Lys residues corresponding to Lys1467 of CdiA^{STEC4} are marked by a red star and rendered in red font. Strains in blue font do not contain *cdiC* in their *cdi* gene clusters.
