# Supplementary material for: Lipidation of Class IV CdiA Effector Proteins Promotes Target Cell Recognition during Contact-Dependent Growth Inhibition
Source: mBio. 2021 Oct 12;12(5):e02530-21. doi: 10.1128/mBio.02530-21 (PMC8510554; doi:10.1128/mBio.02530-21)
Supplement: FIG S7 [file mbio.02530-21-sf007.pdf]

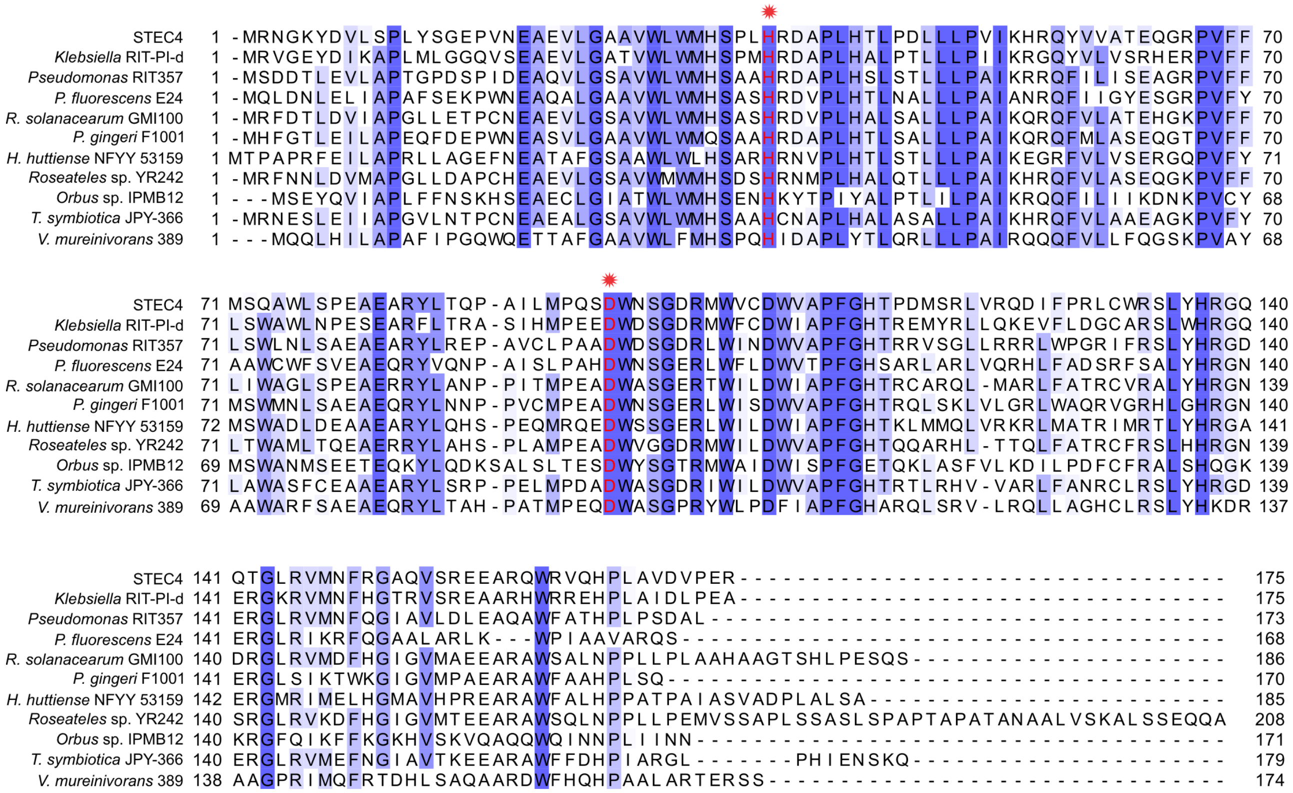

Figure S7. Alignment of predicted CdiC lysyl acyltransferases. Predicted CdiC proteins from the bacterial strains listed in Figure S8 were aligned with Clustal omega and the results rendered using Jalview. Sequences are from the following bacterial strains (NCBI reference identifiers in parentheses): *E. coli* STEC_O31 (WP_001243916.1); *Klebsiella* sp. RIT-PI-d (WP_049840268.1); *Pseudomonas* sp. RIT357 (WP_032887925.1); *Pseudomonas fluorescens* E24 (WP_078827813.1); *Ralstonia solanacearum* GMI100 (WP_011004363.1); *Pseudomonas gingeri* F1001 (WP_177143638.1); *Herbaspirillum huttiense* NFYY 53159 (WP_134221495.1); *Roseateles* sp. YR242 (WP_092949727.1); *Orbus* sp. IPMB12 (WP_166917330.1); *Trinickia symbiotica* JPY-366 (WP_107149767.1); and *Vogesella mureinivorans* 389 (WP_147695852.1). The catalytic dyad corresponding to His37 and Asp107 of $CdiC^{STEC4}$ are marked by a red star and rendered in red font.
