# Supplementary material for: Lipidation of Class IV CdiA Effector Proteins Promotes Target Cell Recognition during Contact-Dependent Growth Inhibition
Source: mBio. 2021 Oct 12;12(5):e02530-21. doi: 10.1128/mBio.02530-21 (PMC8510554; doi:10.1128/mBio.02530-21)
Supplement: TABLE S1 [file mbio.02530-21-st001.docx]

**Table S1. Bacterial strains and plasmids**

| **Strain** | **Description^a^** | **Reference** |
| --- | --- | --- |
| DH5α *pir*^+^ | *endA1 hsdR17 glnV44 (= supE44) thi-1 recA1 gyrA96 relA1 Φ80dlac∆(lacZ)M15 ∆(lacZYA-argF)U169 zdg-232::Tn10 uidA::pir^+^*, Tet^R^ Nal^R^ | Stratagene |
| EPI100 | F^−^ *mcrA* ∆(*mrr-hsdRMS-mcrBC*) φ80d*lacZ∆*M15 *∆lacXcZ*∆M15 *∆lacX recA1 endA1 araD139* ∆(*ara*, *leu*)*7697 galU galK* λ^−^ *rpsL nupG*, Str^R^ | Epicentre |
| MC1061 | F- *araD139 (ara-leu)7679 (lacIPOZYA)X74 galU galK hsdR2 mcrB1 rpsL*, Str^R^ | (1) |
| MFD *pir^+^* | MG1655 *RP4-2-Tc::[∆Mu1::aac(3)IV-∆aphA-∆nic35-∆Mu2::zeo] ∆dapA::(erm-pir) ∆recA* | (2) |
| MG1655 | wild-type *E. coli* K-12 strain | lab collection |
| X90 | *E. coli* F´ *lacI^q^ lac´ pro´/ara ∆(lac-pro) nal1 argE(amb) rif^r^ thi-1*, Rif^R^ | (3) |
| CH2016 | X90 (DE3) ∆*rna ∆slyD::kan*, Rif^R^ Kan^R^ | (4) |
| CH4874 | MC1061 *glmS::*P_BAD_-*cdiC*^STEC4^, Gm^R^ | This study |
| CH5775 | EPI100 ∆*wzb ∆ompC::kan*, Str^R^ Kan^R^ | (5) & this study |
| CH5776 | EPI100 ∆*wzb bamA(∆A672-N681) arfB::kan*, Str^R^ Kan^R^ | This study |
| CH5777 | EPI100 ∆*wzb ∆tsx::kan*, Str^R^ Kan^R^ | (5) & this study |
| CH5806 | EPI100 ∆*wzb ∆waaG::kan*, Str^R^ Kan^R^ | (5) & this study |
| CH5807 | EPI100 ∆*wzb ∆waaQ::kan*, Str^R^ Kan^R^ | (5) & this study |
| CH7175 | EPI100 ∆*wzb*, Str^R^ | (6) |
| CH7367 | MG1655 ∆*wzb* | (7) |
| CH13815 | EPI100 ∆*wzb ∆waaC::kan*, Str^R^ Kan^R^ | This study |
| CH13816 | EPI100 ∆*wzb ∆waaF::kan*, Str^R^ Kan^R^ | (5) & this study |
| CH13817 | EPI100 ∆*wzb ∆waaP::kan*, Str^R^ Kan^R^ | This study |
| CH13818 | EPI100 ∆*wzb ∆waaY::kan*, Str^R^ Kan^R^ | This study |
| CH15163 | MG1655 *glmS::gent*, Gm^R^ | This study |
| CH15164 | MG1655 *glmS::kan*, Kan^R^ | This study |
| **Plasmid** |  |  |
| pKAN | pBluescript derivative with FRT-flanked kanamycin-resistance cassette, Amp^R^ Kan^R^ | (8) |
| pMF19 | expresses the *wbbL* rhamnosyltransferase gene for O16 antigen synthesis, Spm^R^ | (9) |
| pSC189 | mobilizable R6Kγ origin plasmid that carries the *mariner* transposon, Amp^R^ Kan^R^ | (10) |
| pSIM6 | heat-inducible expression of the phage λ Red recombinase proteins on a temperature-sensitive plasmid, Amp^R^ | (11) |
| pTNS2 | carries the *tnsABCD* operon for recombination with sequences cloned onto pUC18R6k-mini*Tn*7T at *att*Tn7, Amp^R^ | (12) |
| pTrc99KX | pTrc99A derivative with 5´-KpnI and 3´-SpeI and XhoI restriction sites, Amp^R^ | (13) |
| pUC18R6k-miniTn7T-Gmr | vector for Tn*7* mediated transposition into the *glmS* locus, Amp^R^ Gm^R^ | (12) |
| pCH296 | pCH450KX::*cdiC*, Tet^R^ | This study |
| pCH405∆ | pACYC184 empty vector derivative, Tet^R^ | (4) |
| pCH450 | pACYC184 derivative that carries *araC* and P*_ara_* promoter, Tet^R^ | (4) |
| pCH1055 | pET21(MCS-)::*cdiBCAI*^STEC4^, Amp^R^ | This study |
| pCH1058 | pET21(MCS-)::*cdiBCA(K1469A)I*^STEC4^, Amp^R^ | This study |
| pCH1061 | pCH405∆::*cdiI*^STEC4^, Tet^R^ | This study |
| pCH1138 | pET21(MCS-)::*cdiBCA(C1243S)I*^STEC4^, Amp^R^ | This study |
| pCH1139 | pET21(MCS-)::*cdiBCA(K1466A)I*^STEC4^, Amp^R^ | This study |
| pCH1140 | pET21(MCS-)::*cdiBCA(K1467A)I*^STEC4^, Amp^R^ | This study |
| pCH1145 | pET21(MCS-)::∆prom-*cdiBCAI*^STEC4^, Amp^R^ | This study |
| pCH4469 | pET21(MCS-)::*cdiBAI*^STEC4^, Amp^R^ | This study |
| pCH4470 | pET21(MCS-)::*cdiBC(H37A)AI*^STEC4^, Amp^R^ | This study |
| pCH4471 | pET21(MCS-)::*cdiBC(D107A)AI*^STEC4^, Amp^R^ | This study |
| pCH4472 | pET21(MCS-)::*cdiBCA(K1467R)I*^STEC4^, Amp^R^ | This study |
| pCH4872 | pUC18R6k-miniTn7T-*cdiC*^STEC4^, Gm^R^ | This study |
| pCH6884 | pET21(MCS-)::*cdiBCA(K1467Q)I*^STEC4^, Amp^R^ | This study |
| pCH6962 | pTrc99KX::*cdiC*, Amp^R^ | This study |
| pCH7391 | pACYCDuet::RBD(K1467Q)^STEC4^, Cm^R^ | This study |
| pCH9674 | pSIM6::*recA*, Amp^R^ | This study |
| pCH13167 | pET21b::*cdiBCAI*^STEC4^, Amp^R^ | This study |
| pCH13508 | pKAN::∆*waaC*, Amp^R^ Kan^R^ | This study |
| pCH13509 | pKAN::∆*waaP*, Amp^R^ Kan^R^ | This study |
| pCH13510 | pKAN::∆*waaY*, Amp^R^ Kan^R^ | This study |
| pCH13581 | pCH450KX::*waaF*, Tet^R^ | This study |
| pCH13582 | pCH450KX::*waaP*, Tet^R^ | This study |
| pCH13658 | pET21(MCS-)::*cdiBAI*^STEC3^, Amp^R^ | (7) |
| pCH14181 | pTrc99KX::*cdiC(H37A)*, Amp^R^ | This study |
| pCH14182 | pTrc99KX::*cdiC(D107A)*, Amp^R^ | This study |
| pCH14473 | pCH450KX::*waaC*, Tet^R^ | This study |
| pCH14508 | pACYCDuet::RBD^STEC4^, Cm^R^ | This study |
| pCH14660 | pACYCDuet::RBD(K1466A)^STEC4^, Cm^R^ | This study |
| pCH14661 | pACYCDuet::RBD(K1467A)^STEC4^, Cm^R^ | This study |
| pCH14662 | pACYCDuet::RBD(K1469A)^STEC4^, Cm^R^ | This study |
| pCH15099 | pACYCDuet::RBD(K1467R)^STEC4^, Cm^R^ | This study |
| pCH15160 | pET21b::*cdiA(V1269-P1589)*^STEC4^, Amp^R^ | This study |
| pCH15268 | pACYCDuet::*cdiA(V1269-P1589)*^STEC4^, Cm^R^ | This study |

*^a^*Abbreviations: Amp^R^, ampicillin resistant; Cm^R^, chloramphenicol resistant; Gm^R^, gentamicin resistant; Kan^R^, kanamycin resistant; Nal^R^, nalidixic acid resistant; Rif^R^, rifampicin resistant; Spm^R^, spectinomycin resistant; Str^R^, streptomycin resistant; Tet^R^, tetracycline resistant

1. **Casadaban MJ, Cohen SN.** 1980. Analysis of gene control signals by DNA fusion and cloning in *Escherichia coli*. J Mol Biol **138:**179-207.

2. **Ferrieres L, Hemery G, Nham T, Guerout AM, Mazel D, Beloin C, Ghigo JM.** 2010. Silent mischief: bacteriophage Mu insertions contaminate products of *Escherichia coli* random mutagenesis performed using suicidal transposon delivery plasmids mobilized by broad-host-range RP4 conjugative machinery. J Bacteriol **192:**6418-6427.

3. **Beckwith JR, Signer ER.** 1966. Transposition of the *lac* region of *Escherichia coli*. I. Inversion of the *lac* operon and transduction of *lac* by phi80. J Mol Biol **19:**254-265.

4. **Garza-Sánchez F, Janssen BD, Hayes CS.** 2006. Prolyl-tRNA(Pro) in the A-site of SecM-arrested ribosomes inhibits the recruitment of transfer-messenger RNA. J Biol Chem **281:**34258-34268.

5. **Baba T, Ara T, Hasegawa M, Takai Y, Okumura Y, Baba M, Datsenko KA, Tomita M, Wanner BL, Mori H.** 2006. Construction of *Escherichia coli* K-12 in-frame, single-gene knockout mutants: the Keio collection. Mol Syst Biol **2:**2006 0008.

6. **Aoki SK, Malinverni JC, Jacoby K, Thomas B, Pamma R, Trinh BN, Remers S, Webb J, Braaten BA, Silhavy TJ, Low DA.** 2008. Contact-dependent growth inhibition requires the essential outer membrane protein BamA (YaeT) as the receptor and the inner membrane transport protein AcrB. Mol Microbiol **70:**323-340.

7. **Ruhe ZC, Subramanian P, Song K, Nguyen JY, Stevens TA, Low DA, Jensen GJ, Hayes CS.** 2018. Programmed Secretion Arrest and Receptor-Triggered Toxin Export during Antibacterial Contact-Dependent Growth Inhibition. Cell **175:**921-933 e914.

8. **Hayes CS, Sauer RT.** 2003. Cleavage of the A site mRNA codon during ribosome pausing provides a mechanism for translational quality control. Mol Cell **12:**903-911.

9. **Feldman MF, Marolda CL, Monteiro MA, Perry MB, Parodi AJ, Valvano MA.** 1999. The activity of a putative polyisoprenol-linked sugar translocase (Wzx) involved in Escherichia coli O antigen assembly is independent of the chemical structure of the O repeat. J Biol Chem **274:**35129-35138.

10. **Chiang SL, Rubin EJ.** 2002. Construction of a mariner-based transposon for epitope-tagging and genomic targeting. Gene **296:**179-185.

11. **Datta S, Costantino N, Court DL.** 2006. A set of recombineering plasmids for gram-negative bacteria. Gene **379:**109-115.

12. **Choi KH, Gaynor JB, White KG, Lopez C, Bosio CM, Karkhoff-Schweizer RR, Schweizer HP.** 2005. A Tn7-based broad-range bacterial cloning and expression system. Nat Methods **2:**443-448.

13. **Beck CM, Morse RP, Cunningham DA, Iniguez A, Low DA, Goulding CW, Hayes CS.** 2014. CdiA from *Enterobacter cloacae* delivers a toxic ribosomal RNase into target bacteria. Structure **22:**707-718.
