# Supplementary material for: Lipidation of Class IV CdiA Effector Proteins Promotes Target Cell Recognition during Contact-Dependent Growth Inhibition
Source: mBio. 2021 Oct 12;12(5):e02530-21. doi: 10.1128/mBio.02530-21 (PMC8510554; doi:10.1128/mBio.02530-21)
Supplement: TABLE S2 [file mbio.02530-21-st002.docx]

**Table S2. Oligonucleotides**

| **Identifier** | **Description** | **Sequence^a^** | **Reference** |
| --- | --- | --- | --- |
| CH2131 | recA-BglII | 5´ - TGC AGA TCT TGT GGC AAC AAT TTC TAC | This study |
| CH2132 | recA-Xma | 5´ - GCG ACC CGG GTG TAT CAA ACA AGA CG | This study |
| CH2260 | mariner-rev-seq | 5´ - CAA GCT TGT CAT CGT CAT CC | This study |
| CH4087 | cdiC-Kpn-for | 5´ - GAA GGT ACC ATG CGT AAC GGG AAA TAT | This study |
| CH4088 | cdiC-Xho-rev | 5´ - AAA CTC GAG TTA TCT CTC CGG CAC ATC | This study |
| CH4174 | cdiC-D107A-for | 5´ - CCG GAT GTG GGT CTG TGC CTG GGT TGC CCC TTT TG | This study |
| CH4175 | cdiC-D107A-rev | 5´ - CAA AAG GGG CAA CCC AGG CAC AGA CCC ACA TCC GG | This study |
| CH4176 | cdiC-H37A-for | 5´ - GAT GCA CTC CCC CCT GGC GCG TGA TGC GCC ACT G | This study |
| CH4177 | cdiC-H37A-rev | 5´ - CAG TGG CGC ATC ACG CGC CAG GGG GGA GTG CAT C | This study |
| CH4195 | waaC-KO-Sac-for | 5´ - TTT GAG CTC GCT TTC ATC AGA ACG TCC GAT G | This study |
| CH4196 | waaC-KO-Bam-rev | 5´ - TTT GGA TCC GTAA CAA TAG CGC GTT GAG TTC TTC C | This study |
| CH4197 | waaC-KO-Eco-for | 5´ - TTT GAA TTC AGG TAA AAC ATG CTA ACA TCC TTT AAA C | This study |
| CH4198 | waaC-KO-Kpn-rev | 5´ - TTT GGT ACC AAC GCC ACT AAC TAT CCC TAT TAG C | This study |
| CH4199 | waaP-KO-Sac-for | 5´ - TTT GAG CTC GCT TTG GCA TCG TTA CCG G | This study |
| CH4200 | waaP-KO-Bam-rev | 5´ - TTT GGA TCC CCA AAG TGT GGC AAG CGG | This study |
| CH4201 | waaP-KO-Eco-for | 5´ - TTT GAA TTC GAG CGA ACA CAA CGC AAA GG | This study |
| CH4202 | waaP-KO-Kpn-rev | 5´ - TTT GGT ACC GGA AAA AAC ATA TTG GCT GGC TG | This study |
| CH4203 | waaP-KO-Sac-for | 5´ - TTT GAG CTC GAA AGG GAT GAC ATT ATT TTT GCC TCG | This study |
| CH4204 | waaP-KO-Bam-rev | 5´ - TTT GGA TCC CCA GTT AAA TGT TAT TTA CGG TAA TAT TTT C | This study |
| CH4205 | waaP-KO-Eco-for | 5´ - TTT GAA TTC CCA CAA TTA CAT GTC TTC ACC AGG | This study |
| CH4206 | waaP-KO-Kpn-rev | 5´ - TTT GGT ACC CCC GAC GGT AAA AGG ACC G | This study |
| CH4207 | waaF-Not-for | 5´ - TTT GCG GCC GCA ATC GCG ACG CAT AAG AGC | This study |
| CH4208 | waaF-Xho-rev | 5´ - TTT CTC GAG TCC GTC AGC TTC CTC TTG | This study |
| CH4209 | waaP-Not-for | 5´ - TTT GCG GCC GCG GAT ATC ATT ACA GGT GG | This study |
| CH4210 | waaP-Xho-rev | 5´ - TTT CTC GAG TTA TAA TCC TTT GAG TTG TGT TCG | This study |
| CH4299 | waaF-Sac-for | 5´ - TTT GAG CTC CCT GCC TGA AGC GAA CTC G | This study |
| CH4300 | waaF-Kpn-rev | 5´ - TTT GGT ACC GCG ATA GCA TAA TCG CCC TGG | This study |
| CH4358 | STEC4-V1328-Nco-for | 5´ - TTT CCA TGG TCA ATG ACC ATT TCA CCA CGG AGC | This study |
| CH4359 | STEC4-P1589-Xho-rev | 5´ - TTT CTC GAG TTA GTG GTG ATG ATG ATG ATG TGG CAC ATT CAC TGC CGG | This study |
| CH4387 | waaC-Kpn-for | 5´ - TTT GGT ACC ATG CGG GTT TTG ATC GTT AAA AC | This study |
| CH4388 | waaC-Xho-rev | 5´ - TTT CTC GAG TTA TAA TGA TGA TAA CTT TTC CAA AAC TGC | This study |
| CH4616 | Tn7R-screen-rev | 5´ - CAC AGC ATA ACT GGA CTG ATT TC | This study |
| CH4647 | STEC4-K1467A-for | 5´ - TAC CGT ATC AGG AAA GCA GGA AAG GAT AAG CA | This study |
| CH4648 | STEC4-K1469A-for | 5´ - ATC AGG AAA AAA GGA GCG GAT AAG CAG GG | This study |
| CH4672 | glmS-for | 5´ - GAG ATG CCG CAC GTT GAG G | This study |
| CH4802 | STEC4-N1229-Eco-for | 5´ - CTG ACG AAT TCC GGT ACC GGG CGA ATC | This study |
| CH4803 | STEC4-S2159-Xba-rev | 5´ - CCT GCA GGG CTT CAA GTC TAG AGT TTC CGG CAG ATT TCG | This study |
| CH4804 | STEC4-S2159-Xba-for | 5´ - GCA AAT CTG CCG GAA ACT CTA GAC TTG AAG CCC TGC AGG | This study |
| CH4841 | STEC4-K1466A-rev | 5´ - GCT TAT CCT TTC CTT TTG CCC TGA TAC GGT AAT AAT AC | This study |
| CH4860 | STEC4-C1243S-Eco-for | 5´ - GAC GAA TTC CGG TAC CGG GCG AAT CTA CGG CGA TGC TGT TGG TGT CTC CGC TGC CAC ATT | This study |
| CH4869 | STEC4-cdiI-Kpn-for | 5´ - TTG GTA CCA TGA TTT TAA ATG ATT TTT TTT TAT TAA TGC TTG C | This study |
| CH4888 | STEC4-K1467R-rev | 5´ - GCT TAT CCT TTC CTC TTT TCC TGA TAC GGT AAT AAT AC | This study |
| CH4964 | STEC4-P77-Nco-rev | 5´ - GCT GCT GAT CCT GAG GTG CGC CAT GG | This study |
| CH4991 | STEC4-V1269-Nhe-for | 5´ - TTT GCT AGC GTA CAG ACC CTG AAT AAC CGG | This study |
| CH5186 | STEC4-K1467Q-rev | 5´ - GCT TAT CCT TTC CTT GTT TCC TGA TAC GGT AAT AAT AC | This study |
| CH5507 | waaG-KO-Sac-for | 5´ - TTC GAG CTC AGT AAA TAG CTG ACT TAT GGA TG | This study |
| CH5508 | waaG-KO-Kpn-rev | 5´ - TCA GGT ACC GTT TGA TAA TGC TTC TGG AAA TC | This study |
| CH5509 | waaQ-KO-Sac-for | 5´ - GAT GAG CTC CAA CAG CGA GGC | This study |
| CH5510 | waaQ-KO-Kpn-rev | 5´ - TTA GGT ACC ATA GTT GCT ACA CGT GCG | This study |
| ZR253 | cdiB J2ELL3-Asc-rev | 5´ - TTT GGC GCG CCA GAA CGT CAT ATT TCC CGT TAC G | This study |
| ZR258 | J2ELL3 CDI-Not-for | 5´ - TTT GCG GCC GCT CAG GAG ACT GAG TTT CCT GAT G | This study |
| ZR259 | J2ELL3 CDI-Xho-rev | 5´ - TTT CTC GAG CAC AAG CTC AGA CAG CGC | This study |

*^a^*Restriction endonuclease sites underlined
